# Identifying Factors Influencing Attention in Adolescents with a Co-Created Questionnaire: A Citizen Science Approach with Secondary Students in Barcelona, Spain

**DOI:** 10.3390/ijerph18158221

**Published:** 2021-08-03

**Authors:** Florence Gignac, Caterina Solé, Jose Barrera-Gómez, Cecilia Persavento, Èlia Tena, Mónica López-Vicente, Jordi Júlvez, Jordi Sunyer, Digna Couso, Xavier Basagaña

**Affiliations:** 1 Barcelona Institute for Global Health (ISGlobal), Barcelona Biomedical Research Park (PRBB), 08003 Barcelona, Spain; florence.gignac@isglobal.org (F.G.); jose.barrera@isglobal.org (J.B.-G.); cecilia.persavento@isglobal.org (C.P.); jordi.sunyer@isglobal.org (J.S.); 2 Department of Experimental and Health Sciences, Universitat Pompeu Fabra, 08002 Barcelona, Spain; 3 CIBER Epidemiología y Salud Pública, 28009 Madrid, Spain; 4 Departament de Didactica de les Ciencies, Facultat d’Educacio, Edifici G5, Campus de la UAB, Universitat Autònoma de Barcelona, 08193 Bellaterra, Spain; Caterina.Sole@uab.cat (C.S.); elia.tena@uab.cat (È.T.); Digna.Couso@uab.cat (D.C.); 5 Department of Child and Adolescent Psychiatry/Psychology, Erasmus University Medical Centre-Sophia Children’s Hospital, P.O. Box 2060, 3000 CB Rotterdam, The Netherlands; m.lopez-vicente@erasmusmc.nl; 6 Institut d’Investigació Sanitària Pere Virgili (IISPV), Hospital Universitari Sant Joan de Reus, 43204 Reus, Spain; jordi.julvez@urv.cat

**Keywords:** adolescents, attention, citizen science, secondary education, questionnaire design, public health

## Abstract

Studies on factors that can influence attention in healthy adolescents are recent and focus on recurrent topics. Students’ contribution to public health research often revolves around collecting data but rarely around creating data collection instruments. The ATENC!Ó project reunited secondary students and scientists to create a questionnaire including factors that students thought could affect their attention. We conducted a cross-sectional study to assess whether the factors included in this questionnaire had an effect on attention in adolescents. A total of 1667 students (13–16 years old) from 28 schools in Barcelona performed a validated attention test and answered the questionnaire. The response speed consistency (attentiveness), expressed as hit reaction time standard error (HRT-SE, in ms), was used as the primary outcome. Analyses were conducted using conditional linear regression with school as strata, adjusted for sociodemographic characteristics and further stratified by gender and maternal social class. Some factors showed a negative influence on attention, including taking medication and not reading regularly. We found a significant 14.3% (95% confidence interval: 3.4%, 25.3%) higher median of HRT-SE (increase inattentiveness) among students who reported not having a good relationship with classmates. Students’ input into research is relevant for advancing the knowledge production in public health.

## 1. Introduction

Over the last decade, there has been an increased application of participatory approaches in public health studies, including participatory action research, community-based participatory research, popular epidemiology and citizen science (CS) [1,2]. CS broadly refers to the general public engagement in different research practices generating scientific knowledge [3]. CS in public health offers an approach for researchers to better include lay knowledge into scientific knowledge and to further engage stakeholders such as civil society organizations and educational communities in the research process [2]. 

Prior to the recent growing enthusiasm for CS, the educational community was already, albeit rather modestly, engaged in public health research studies [4,5]. Nowadays, CS projects in schools (pre-university levels) are gaining in popularity and have become an opportunity to further engage young students in genuine science, technology, engineering, and mathematics (STEM) practices [6,7]. CS can help teachers and students to better connect with real-world science and have an authentic access to science in action [8]. In fact, students who participate in multiple stages of the scientific process can appreciate a more accurate and realistic view of science and its nature. However, CS projects conducted in schools to investigate public health issues mainly engage students to collect, interpret and report data or to be involved in the development of interventions [9,10,11,12,13,14]. We found only a few CS projects that engaged students in the creation of data collection instruments or similar tools (e.g., screening tools) for public health research [15,16].

In this paper, we present the ATENC!Ó Project (https://projecteatencio.cat/) (accessed on 8 June 2021), where secondary students in Barcelona (Spain) co-created with scientists a questionnaire for identifying potential factors that could influence attention performance in adolescents. Our aim is to assess whether the factors included in this questionnaire are associated with attention performance in adolescents. To do so, we invited secondary students to participate in an epidemiological experiment in which they had to complete a validated attention test and to answer their questionnaire. A parallel objective of this paper is to gauge the benefits of co-creating a data collection instrument and of engaging the educational community for the democratization and advancement of public health research.

## 2. Theoretical Background

### 2.1. Adolescent Cognitive Development

During adolescence, the brain continues to refine and undergo important morphological and functional transformations [17]. Among the last areas of the brain to mature are the frontal lobes, which are known to mediate essential cognitive processes including attention [18]. Hence, adolescence represents a period in which the development of cognitive functions is highly vulnerable to biological, social and environmental perturbations [19]. 

Attention is a complex construct in neuropsychology. The term encompasses several subfunctions such as the preparedness for and selection of specific stimuli of the environment [20], the capacity to inhibit prepotent responses and the ability to focus for a long period of time [21]. Neuropsychological and neuroimaging studies have suggested that attention is divided into three networks, that is, three anatomical areas carrying out the functions of alerting, orienting and executive attention [22]. Posner and Rothbart [23] defined alerting as the ability to maintain a state of high vigilance to incoming stimuli and orienting as the ability to select information from sensory signals. Researchers have often used the term executive attention to refer to the ability to monitor and resolve conflict among responses.

Poor attention in adolescents can be detrimental to their academic performance and socioemotional development [24]. Plus, attentional impairments are characteristic of several disorders (e.g., schizophrenia and attention-deficit/hyperactivity disorder (ADHD)) [23]. Considering that all these individual consequences can yield negative societal and economic impacts later in life, researchers have recently advocated for conducting more studies on adolescents’ development [25].

### 2.2. Factors Associated with Attention

Research that examines protective and risk factors associated with attention in healthy adolescents is not extensive. To date, most of the studies on attention have been carried out on young children, adults or clinical populations (e.g., children diagnosed with ADHD) [26], investigating other outcomes interrelated with attention such as cognitive functions (e.g., working memory) or measures of school performance (e.g., grades). 

The literature on factors influencing attention in healthy adolescents is relatively recent and touches upon recurrent topics. Most of the studies have been centered on the negative effects of television and video games exposure [27], frequent use of mobile phone [28], alcohol consumption [29], tobacco smoke exposure and marijuana use [30,31]. Gender and parental socioeconomic status (SES) are also factors that have been associated with adolescents’ attention performance, but further research is needed [26,32].

Furthermore, previous research has pointed out positive effects of physical activity on attention in adolescents [33]. Some studies have explored the association of dietary patterns and of specific food components with attention in adolescence [34,35,36]. Early life factors such as birth weight and exclusive breastfeeding have also been associated with attention in adolescents [37]. 

Overall, further research on factors affecting attention in adolescents is needed. Usually, to find such factors, researchers develop questionnaires to collect data on SES, behaviors, medical history, etc., and the questions are often selected based on their knowledge and previous literature. Input from the subjects of research (i.e., the adolescents) based on their knowledge and personal experiences could lead to the identification of potential protective and risk factors, some of which may have been overlooked by researchers.

## 3. Methods

### 3.1. Project Overview and Recruitment

The ATENC!Ó project was the result of a partnership between the health research institution ISGlobal (Barcelona Institute for Global Health), the education research center CRECIM (Centre for Research in Science and Mathematics Education of the Autonomous University of Barcelona) and 32 secondary schools in Barcelona and its surroundings, including more than 2000 students between 13 and 16 years old (https://projecteatencio.cat/) (accessed on 8 June 2021). The general aim of the project was to involve students, teachers and scientists in a CS project linked to an environmental health experimental study on the possible influence of air pollution on attention performance in adolescents. The goal of this multidisciplinary collaboration was to advance public health science in an open manner, improve students’ scientific literacy and empower the educational community as contributors to real-world research. To this end, a teaching–learning sequence (i.e., ordered academic activities) was designed and integrated into the schools’ curriculum and an experimental study was conducted [38,39]. The teaching–learning sequence led to the co-creation of a questionnaire identifying factors that could affect attention in adolescents (Section 3.2) to be applied in the experimental study (Section 3.3). Here, we present the results of a cross-sectional analysis to assess whether the factors included in this questionnaire had an effect on attention in adolescents.

We presented the ATENC!Ó project and its study protocol during teacher training sessions open to all science teachers from all secondary schools of the Barcelona metropolitan area. Teachers interested in participating in the co-creation activity and/or the experimental study signed up in an online form. Whereas all students could participate with their classmates on the design of the questionnaire, only students with an informed consent form signed by them and their parents or legal guardians were able to participate in the attention test. There were no exclusion criteria for participating in the attention test. The study was approved by the Parc de Salut Mar Clinical Research Ethics Committee (approval number: 2018/7968/I).

### 3.2. Co-Creation Process to Develop a Data Collection Instrument

**Figure 1:**
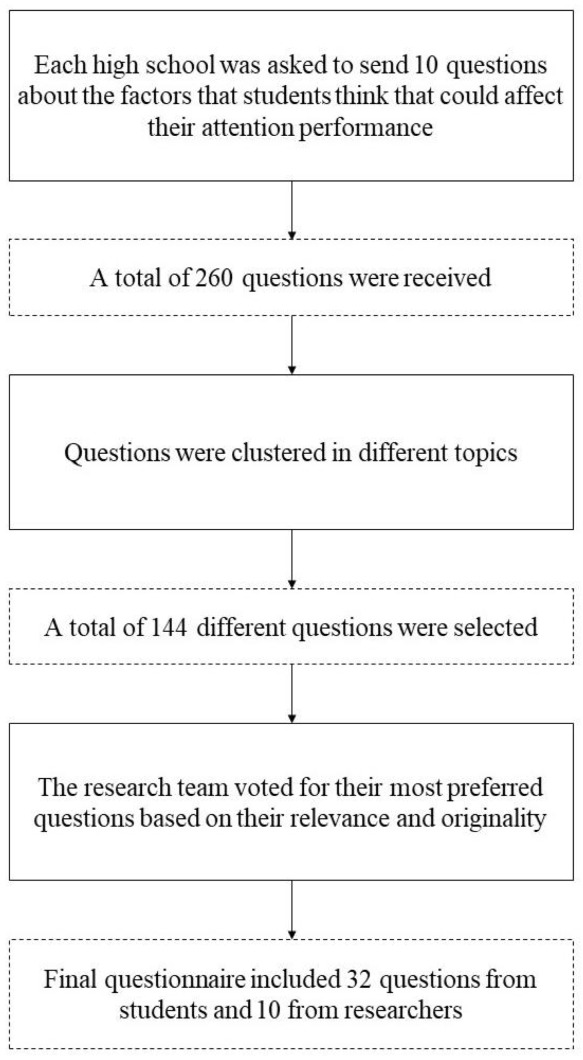
Co-creation process of the questionnaire.

We conducted the co-creation of the questionnaire from September to December 2018 and it consisted of three phases: (1) Question formulation, (2) Question grouping, and (3) Question rating (Figure 1). First, we played a short introduction video to all students from the 32 schools explaining the concept of attention—as a skill to perform a task without distractions—and the experiment setting (see Section 3.3). Next, in groups, we asked the students to propose factors that they thought could affect their attention. Students had to formulate these factors into actual questions for the questionnaire. We received a total of 260 questions (26 class groups from 12 secondary schools developed 10 questions). After removing repeated or similar questions, we kept a total of 144 different questions formulated by the students. 

In the third phase, we asked each member of our scientific research team to rate the questions on a scale from 1 to 4 according to two evaluation criteria: relevance and originality. The questions were rated as highly relevant when the evaluators considered that an association between attention and the factor was highly plausible, whereas questions rated as highly original referred to factors scarcely studied in the literature. We selected and included in the final version of the questionnaire the questions with the highest ratings both for relevance and originality. Our research team was gender-balanced and multidisciplinary, including not only experts in the topic investigated (neuropsychology, epidemiology and statistics), but also researchers in science education. This last phase led to the creation of the final questionnaire, including 32 original questions suggested by the students, to which we added ten general sociodemographic questions (Table 1). We tried to keep the questions and choices of answers exactly as they were written by the students. This explains why moderate answers were different in Q1 (Family relationship quality) and Q2 (Classmates relationship quality). We grouped the 32 questions into six different categories: “Social relationships and interactions”, “Psychological and physical health”, “Eating habits and addictive-substances consumption”, “Use of technology and other habits”, “Other personal conditions during the test” and “Perceived classroom conditions during the test”.

### 3.3. Experimental Study Design and Data Collection

The experimental study was a randomized controlled trial conducted from January to June 2019 and aimed to assess the potential impact of using air cleaner devices (intervention) for a period of 1.5 h in secondary schools on the attention processes of adolescents [39]. During this trial, students were asked to complete different activities on a laptop. One of them was the Attention Network Task-Flanker Task (ANT), to be completed before and after the intervention [40]. They were also required to do an intelligence test (PMA-R test, Primary Mental Aptitudes-Reasoning) [41]. The questionnaire designed by the students was administered via Qualtrics Survey Software (Qualtrics. Provo, UT, USA) at the end of the experiment and data were collected anonymously.

In the present study, we only used the results from the ANT at baseline and PMA-R, which could not be affected by a potential effect of the air cleaners. The ATENC!Ó trial was registered at the US National Institutes of Health (ClinicalTrials.gov) (accessed on 8 June 2021) #NCT03762239.

### 3.4. Attention Testing

ANT is a widely used neuropsychological tool that measures the three networks of attention, and its validity is supported by studies using neuroimaging and with large child cohorts [40,42,43]. In this test, a row of five arrows appears on the computer screen, either above or below a fixation point and the fixation point is followed by a cue. There can be no cue, a center cue, a double cue (alerts about the upcoming target but not on its location), or a spatial cue (alerts about the upcoming target as well as its location). When the row appears after the cue, the student has to use the arrow keys from the keyboard to indicate as fast as possible if the central arrow (target) was pointing to the left or to the right. The flanker arrows are either pointing in the same (congruent) or opposite (incongruent) direction than the central arrow. In our study, the students were presented with four experimental blocks of 32 trials for a duration of approximately 15 min.

The ANT provides measurements on six outcomes related to attention. The response speed consistency is calculated as hit reaction time standard error (in milliseconds) for correct responses (HRT-SE). A lower HRT-SE indicates consistent reaction times, and thus, a good attention performance [44]. The impulsivity score is computed as the number of incorrect responses (responses made in the opposite direction to the direction of the target arrow). The selective attention score is computed as the number of omission errors (failure to respond to the stimulus). The alerting score is computed by subtracting the median reaction time (RT) in milliseconds for double cue from median RT for the no cue condition (calculations performed after removing the incongruent trials). The orienting score is computed by subtracting the median RT in milliseconds for spatial cue from the RT for central cue (calculations performed after removing the incongruent trials). The conflict score or executive attention is calculated by subtracting the median RTs of the congruent from the median RTs of the incongruent trials (across cue conditions) [44]. Low scores in those six outcomes indicate efficient attention performance.

### 3.5. Data Analysis

We performed descriptive, bivariate and multivariable analyses to analyze the data. We excluded students from the analysis when their ANT test had a low accuracy, that is, when the number of correct responses was lower than 70%. Sociodemographic characteristics of secondary school students were described using percentages for categorical variables and medians and interquartile range (IQR) for continuous variables. The following variables were summarized: gender, age, country of birth, intelligence PMA-R score (total of correct responses), poverty, maternal and paternal occupational status as proxy of social class and education level. We also calculated the median and IQR of the primary outcome of interest (HRT-SE), stratifying by the sociodemographic characteristics of the study population. We applied the Mann–Whitney U-test or Kruskal–Wallis to compare the median of HRT-SE across categories of the participants’ characteristics. To assess the relationship between HRT-SE and age and PMA-R score, we computed Spearman’s non-parametric correlation coefficients.

We first fitted simple linear regression models to assess crude associations between each of the 32 factors and each of the ANT outcomes. This analysis was limited to students with complete information on the sociodemographic variables. Then, we fitted a conditional linear regression using school as strata to assess adjusted associations, which allowed to control for conditions in which the ANT test was administered, such as day of the week, time and weather. The model included all variables that showed a significant crude association and was further adjusted for gender, age, country of birth, PMA-R score, poverty and maternal occupational social class. This analysis was also limited to students with complete information on the variables included in the model. The four continuous variables were categorized according to recommendation guidelines for hours of sleep (“Less than 8 h”, “Between 8 h and 10 h” and “More than 10 h”) and for weekly exercise hours (“Less than 7 h” and “7 h or more”) [45,46], and based on previous literature for eating frequency (“Less than 4 times”, “4 times or more”) and for hours of bad news received (“Less than 12 h”, “Between 12 h and 24 h” and “More than 24 h”) [47,48]. HRT-SE was log-transformed to achieve normality. Therefore, the estimated associations were expressed as the relative (percent) changes in the median of the HRT-SE [49]. For untransformed secondary outcome variables, associations were expressed, as usual, as additive changes in the mean of the outcomes.

Moreover, we conducted a stratified analysis by gender and by maternal occupational social class and tested the interactions, since some studies have demonstrated gender and parental social class differences in cognitive tasks [26,32]. Sensitivity analysis comprised the same methodology using class groups as random effect or using secondary attention outcomes including impulsivity, selective attention, alerting score, orienting score and conflict score. Statistical significance was set at *p* < 0.05. All analyses were conducted using Stata 16.1 (StataCorp, College Station, TX, USA). The dataset is available for download and free use through the file repository Zenodo [50].

## 4. Results

A total of 1747 students from 28 secondary schools completed the attention test. A total of 80 students were excluded due to a low accuracy in the ANT test (*n* = 59) or not answering the questionnaire (*n* = 21). Hence, the final sample size included 1667 students. Table 2 presents a summary of students’ sociodemographic characteristics. The median age of students was 14.8 years (IQR = 14.6–14.9 years) and 50.3% of the sample were girls. Up to 85.5% of the students were born in Spain and appeared to perform better in the attention test (lower HRT-SE) than immigrant students. A total of 7.2% of the adolescents reported struggling to afford basic needs (poverty).

The frequency distribution of responses for each category of all the 32 questions/factors in the questionnaire can be found in Table S1, which also shows the assessment of the crude associations between HRT-SE and the factors. The complete-case analysis sample included 1658 students. Regarding factors related to social relationships (Category 1), students who reported being in a not-so-good relationship with classmates, in a conflict with peers and family, and in love presented a higher HRT-SE (i.e., they were less attentive). As for factors related to psychological and physical health (Category 2), students showed higher HRT-SE when self-rating their health as bad, taking medication and when having pain during their menstruations. For the factors related to the eating habits and addictive-substances consumption (Category 3), we observed a higher HRT-SE among students who did not have breakfast before the experiment, who usually ate less than four times a day or who reported consuming regularly or occasionally tobacco, alcohol, marijuana or energy drinks. In the domain of technology and other habits (Category 4), students who reported using their mobile phone less frequently had lower HRT-SE, whereas students who reported keeping active the notifications on their mobile phone while studying showed poorer attention performance. Students who considered themselves as regular readers had a lower HRT-SE. Students who reported that they were doing physical exercise seven hours or more per week had lower HRT-SE results. When looking at personal conditions before and during the test (Category 5), HRT-SE was higher among students who needed to go to the toilet or who recently received bad news. Finally, when there was noise in the classroom (Category 6), attention performance in students was better. In comparison with HRT-SE, secondary attention outcomes were significantly associated with fewer factors (Supplementary Table S2). In general, those significant relationships were in the same direction as we found with HRT-SE.

**Figure 2:**
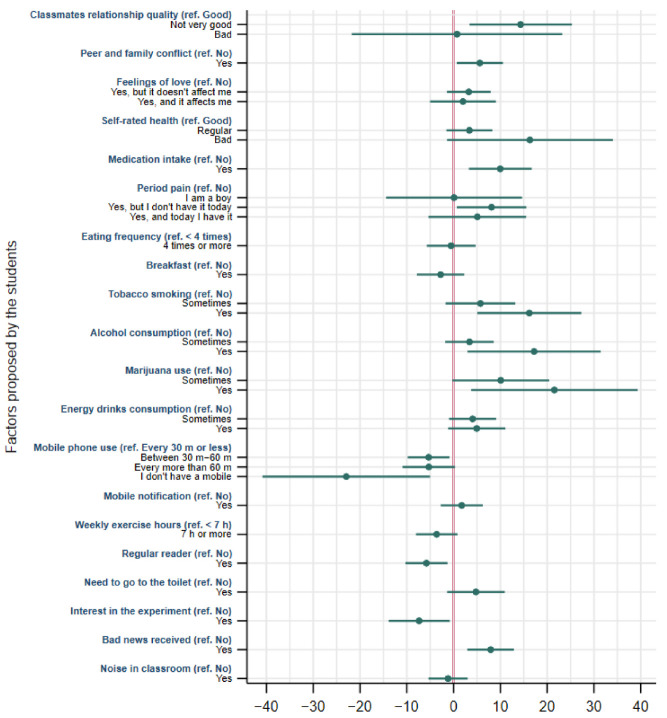
Percent change (95% confidence interval (CI) in attentiveness (HRT-SE)) among factors reported by the students in the co-created questionnaire with a *p*-value < 0.05 in the bivariate analysis (*n* = 1658).

Figure 2 shows the results of a multivariable model including all the variables that showed a crude association (see Supplementary Table S3 for more details). After controlling for gender, age, country of birth, PMA-R score, maternal occupational social class and poverty, we found a 14.3% (95% confidence interval (CI): 3.4%, 25.3%) higher median of HRT-SE in students who reported being in a not-so-good relationship with classmates and a 5.6% (95% CI: 0.7%, 10.5%) higher median in students who were in a conflict with peers or family. As for factors in psychological and physical health, we found higher medians in students who were taking prescribed medications (10%, 95% CI: 3.3%, 16.7%) and who have pain during their periods, but not during the day of the test (8.1%, 95% CI: 0.7%, 15.6%). Regarding addictive-substances consumption, we observed higher medians HRT-SE in students who reported smoking tobacco (16.2%, 95% CI: 5.1%, 27.3%), consuming alcohol (17.2%, 95% CI: 3%, 31.5%) and using marijuana (21.5%, 95% CI: 3.8%, 39.2%). Moreover, we found lower medians among adolescents who reported not having a mobile phone compared to those using it every half hour or less (−22.9%, 95% CI: −40.9%, −5%), even though they represented less than 1% of the students; and among adolescents who considered themselves as regular readers (−5.8%, 95% CI: −10.3%, −1.3%). We also observed a lower median in students who had an interest in doing the experimental study (−7.4% (95% CI: −13.9%, −1%)) and a higher median in students who received recent bad news (−7.9% (95% CI: 2.9%, 12.9%)). When using class groups as strata instead of school, we obtained similar results (Supplementary Table S4).

We did not detect significant interactions by gender (Supplementary Table S5). However, the stratification of the model by the gender of the adolescents showed important differences. Most of the significant factors previously reported in Figure 2 were found only in girls. These included relationship quality with classmates, peer and family conflict, marijuana use, regular reader, interest in the experiment and bad news received (Supplementary Table S5). In boys, significant associations were limited to the factors of medication intake, tobacco smoking, alcohol consumption and frequency use of the mobile phone. We did not detect significant interactions by maternal occupational social class (Supplementary Table S6). However, after stratification of HRT-SE by maternal occupational social class, we observed a higher increase in the medians of HRT-SE in students who reported smoking tobacco, consuming alcohol and marijuana among those whose mothers’ occupational social class was categorized as “Other” (i.e., housewife, unemployed or retired) (Supplementary Table S6). Moreover, students whose mothers occupied a highly skilled job had a 14.2% (95% CI: 5.6%, 22.9%) higher median of HRT-SE when they felt in love and students whose mothers occupied a non-manual job had a 38.0% (95% CI: 3.8%, 72.2%) higher median when rating their health as bad.

**Figure 3:**
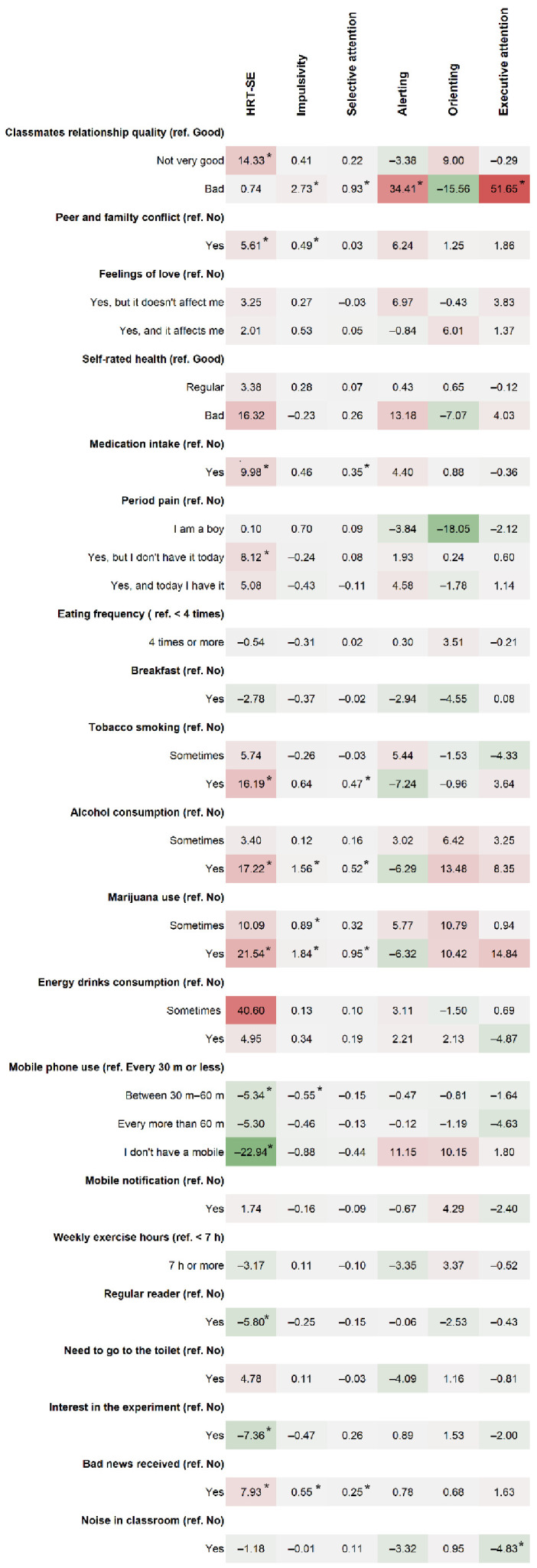
Heatmap of changes for primary and secondary attention outcomes. Supplementary Tables S3 and S7 provide the specific 95% confidence interval and *p*-values. Lower change HRT-SE (percent) impulsivity (count), selective attention (count), alerting score (ms), orienting score (ms) and executive attention score (ms) indicate better attention performance. Significant changes (*p*-value < 0.05) are indicated with a (*).

Adjusted associations when considering secondary attention outcomes, including impulsivity, selective attention, alerting, orienting and executive attention scores resulted in a smaller number of significant associations and with smaller sizes of the point estimates in comparison with the analysis for HRT-SE (Figure 3 and Supplementary Table S7). While there was a nonsignificant small median increase of HRT-SE in students qualifying their relationship with their classmates as bad (0.74%, 95% CI: −21.8%, 23.3%), we found significant higher means of alerting (34.4 ms, 95% CI: 5.4 ms, 63.4 ms) and executive attention scores (51.7 ms, 95% CI: 27.7 ms, 75.6 ms).

## 5. Discussion

Our study shows the relevancy of involving students (non-professional scientists and subjects of the study) in the development of a questionnaire for public health research. In fact, our findings suggest that students proposed factors that were indeed associated with attention test scores, and some of them are understudied factors.

We found that adolescents who were in a peer and family conflict and who perceived their social relationship with classmates as not so good were less attentive than their peers. Some studies have assessed the influence of similar topics about problematic social relationships such as peer victimization and negative emotions on attention in adolescents, but further research is needed to understand their mechanisms [51,52]. Substantial neuroimaging-based evidence has also suggested that adolescents’ neurodevelopment is sensitive to social contexts (e.g., peer interactions) [53]. Also, we found that taking prescribed medication worsened attention performance. This would need further investigation since drugs have different pharmacological properties. Some drugs can improve cognitive function (e.g., nootropics), have no effect on cognition (e.g., anticholinergic medications) [54], or can induce cognitive impairments (e.g., antiepileptic drugs) [55].

Furthermore, adolescents having received bad news or who were lacking interest in doing the experiment presented lower attention. One could link these factors as negative emotional stimuli. These are relevant factors as they dive into an existing complex research on the interactions of emotion and attention, since emotion is able to bias attention and attention to modulate emotional processing [56]. Moreover, in our study, being a regular reader appeared to improve attention, but this finding should be interpreted with considerable caution. Reciprocally related, attention and reading skills have a relationship that encompasses other numerous cognitive abilities such as intelligence quotient, which makes it complex to study their influences [57]. Our analyses, though, were adjusted for PMA-R score, which is a proxy for intelligence, and therefore, they suggest that there is an association between reading and attention that is independent of intelligence. Plus, adolescents with attention problems can often experience difficulties in reading, and as this becomes a hard task for them, they are less tempted to read on a regular basis. Finally, students reported factors such as mobile phone and addictive substance use, which are more commonly studied in public health research, and indeed, our results appeared consistent with the previous literature [28,29,30,31].

When we stratified these analyses according to gender, a number of associations were stronger among female students, suggesting that girls may be more susceptible to the factors included in this study that increase or decrease attention. Gender differences in adolescents have been demonstrated in a number of cognitive domains [58,59]. Studies assessing the effect of similar factors as those proposed by the students observed also some gender differences. For instance, Noorbakhsh et al. [60] found that girls may experience a stronger neurocognitive impairment on working memory than male adolescents after using marijuana. Ramos-Loyo et al. [61] found that emotional contexts may exert a distracting effect on attention in both male and female adolescents, and the latter seemed to spend more time on stimuli processing. Also, considering that girls tend to place a higher value on social goals compared to boys [62], this could explain why, in our study, factors more related to negative affective contexts, including classmate relationships quality, peer and family conflict and bad news received, were exerting a stronger effect on female adolescents.

We also observed that adolescents who were born in Spain seemed to perform better than immigrant adolescents. Immigration background is indeed a sociodemographic factor that can impose risks on the cognitive development of adolescents, and there are several factors that could mediate the relationship between migration and cognitive function [63,64,65]. For instance, in comparison to native adolescents, immigrant adolescents experience additional challenges that co-occur with their cognitive development such as acculturation and discrimination [66]. In Spain, immigrant adolescents tend to report worse mental health than native adolescents and there is a general academic performance gap between immigrant and native secondary students, where immigrant students tend to perform worse than native students [67,68]. Moreover, it is possible that the lack of language proficiency of the immigrant students affected their performance on the attention test. In fact, some of the participating schools were regrouping several adolescents with native languages other than Spanish or Catalan, the two languages that were used to give the instructions and conduct the experiment. Thus, we could suggest that the non-native language status had an adverse effect on attention performance.

Considering that we found a number of factors influencing attention performance and the fact that the latter is related to the optimal functioning of other cognitive abilities raises an important question about the quality of the scores derived from cognitive tests conducted in schools. Indeed, evidence from education research studies have shown substantial performance gaps between students in different motivational conditions [69]. Given that students’ lack of motivation or other emotional factors can impede their performance in the cognitive tests (as we found in our study), the interpretation of the results of such tests may be inaccurate since the latter may not represent the optimal cognitive abilities of those students. Hence, future research applying cognitive tests should take into account more of these types of factors.

Although we did not find associations between some of the factors and attention, there remains a paucity of research on them, highlighting a relevant contribution from the students. For example, while several studies have indicated the positive effect of breakfast on other cognitive functions (e.g., working memory) and academic performance in adolescents [70], studies looking at the effect of breakfast on adolescents’ attention are less frequent and remain unclear [71]. Moreover, the time of the day [72], effects of sleep deprivation [73], feelings of love [74], mobile notifications while doing other tasks are not thoroughly investigated topics in adolescent cognitive health research [75].

In this study, most analyzed factors associated with attention, even after controlling for sociodemographic characteristics and intelligence, are modifiable. Thus, although causality cannot be inferred from our study, these findings have important implications for public health interventions targeting the school setting. Our results could be used to promote healthy habits to improve attention and ultimately learning and academic achievement among students.

Beyond the abovementioned findings, the ATENC!Ó project is a good example of the mutual benefits of incorporating public health CS into secondary education. By giving the adolescents a triple role (as researchers, study subjects and students), this collaborative process enabled responding to the scientific research objectives. In fact, other studies in public health have shown that questionnaire development framed as an open process helps in generating new ideas in accordance with the knowledge of the target population (here, the adolescents), while remaining in line with the researchers’ objectives [15,76]. This process has also an educational value where students can learn the importance of designing data collection instruments in scientific research. Yet, to expand the role of the students as co-researchers, we would recommend that future public health studies interested in replicating this co-creation process also involve the students in censoring the questions and in analyzing the data, which we did not do in our project. Needless to say, however, this collaboration can be challenging in secondary schools as it can require an important engagement in time and effort from the students during a full academic year. Moreover, we believe that co-designing a questionnaire and linking the results to validated computerized tests to measure cognitive outcomes can be relatively easily replicated by educational communities. Indeed, more and more validated computerized tools assessing different aspects of cognitive and mental health are made available [77]. In addition, it may be useful to replicate these results in the full adolescence age range of 10–19 years (the range of 13–16 years was studied here). This could allow for studying the factors of attention in early and mid-adolescence stages and developing a large-scale school research project. It is important to note, however, that the reliability and validity of the co-created questionnaire were not tested, since the goal of the present study was not to develop a questionnaire to be applied for future research, but instead, to inspire other researchers to develop a similar collaborative process. Performing such tests would have required revising and eliminating items that were directly suggested by the students and identifying with them the dimensions they aimed to measure with these questions. Considering that assessing the reliability and validity is highly important to ensure the integrity and quality of a data collection instrument, further studies could shed more light on how to make such assessments while maintaining collaboration with the subjects of research.

The main strengths of this study included the use of an objective and validated measure of attention and a large sample size from many secondary schools in the Barcelona metropolitan area. Moreover, the methods are easily reproducible for researchers interested in conducting a similar study in other settings with students. Additionally, involving students rather than the general public in the formulation of the questions can be sometimes more beneficial for researchers since students have different points of view and levels of interest and understanding towards the scientific topic [78,79]. However, from a CS perspective in which participation often comes from the people who want to volunteer in research activities, we acknowledge that in our study students did not participate voluntarily since the activity was presented as a normal school task. Nevertheless, this helped us to avoid having a skewed profile of participants of only interested people as it often happens in CS projects and avoid selection bias [80]. Even if the activity was not voluntary, we believe this research process can still be qualified as CS considering that non-professional scientists gave their inputs to the design of a scientific research tool.

Some limitations should also be mentioned. First, the cross-sectional nature of our study makes it difficult to infer causality. Nevertheless, the significant associations found and the potential factors suggested by the students can surely inspire new hypotheses for addressing more in-depth research questions or socioneurobiological mechanisms. Second, due to the observational nature of this study design, the risk of residual confounding cannot be ruled out. However, to address this limitation, we included a wide range of potential confounders and conducted sensitivity analyses. Third, responses to the questionnaire were entirely self-reported, and thus, could have led to various biases affecting the results. For instance, asking adolescents to evaluate their behaviors with a self-reported questionnaire is susceptible to a social desirability bias [81]. A tendency to overreport favorable behaviors or to underreport negative ones can happen even if the questionnaire was anonymous. In addition, the fact that a high number of students did not know the education level of their parents or their exact job might have affected the accuracy of the data.

## 6. Conclusions

One key aspect of the ATENC!Ó project was the co-creation of a questionnaire to identify factors that could affect attention in adolescents. We found that students proposed factors that are not extensively studied, and in some cases, those factors appeared to influence attention. Many of these factors are modifiable, such as the quality of social relationships with classmates, reading habits and mobile phone use frequency. Thus, our findings could support the development of public health measures at schools targeting those factors to improve attention in students. Since attention processes are known to be highly related with learning abilities, such initiatives may play a role in ensuring a more favorable learning process and greater academic achievement in adolescents. Overall, our results suggest how valuable it is to involve secondary school students in the creation of a data collection instrument for public health research. This involvement, when carefully planned, contributes not only to democratizing scientific research, but also to advance the production of knowledge in health. As well, we shared the preliminary results of this study with the students during a public event and we sent a reader-friendly report on the final results to all participants. Both the results and the methods used in this study provide future directions for research into adolescent attention development and for participatory practices in public health research carried out in secondary schools.

## Figures and Tables

**Table 1:** Items of the co-created questionnaire.

Category	Question	Variable Name	Answer Option
**Category 1: Social relationships and interactions**	Q1. How is your relationship with your family?	Family relationship quality	Good/Stable/Bad
	Q2. Your relationship with your classmates is	Classmates relationship quality	Good/Not very good/Bad
	Q3. Do you suffer from bullying?	Bullying victimization	No/Yes
	Q4. Have you been in a conflict with someone recently?	Peer and family conflict	No/Yes
	Q5. Are you in love?	Feelings of love	Yes, and it affects me/Yes, but it doesn’t affect me/No
**Category 2:** **Psychological and physical health**	Q6. How are you feeling today in terms of health?	Self-rated health	Good/Regular/Bad
	Q7. Have you taken any medication today?	Medication intake	No/Yes
	Q8. If yes, do you think the medication was affecting you during the attention test?	Medication effect	No/Yes
	Q9. Did you have to do an important mental or physical effort before doing the attention test?	Mental or physical effort	No/Yes
	Q10. Are you going through a significant event in your life?	Significant life event	No/Yes
	Q11. When you are in your period, do you feel uncomfortable?	Period pain	No/I am a boy/Yes, and today I have it/Yes, but I don’t have it today
	Q12. Do you suffer from a disease that makes you feel chronic pain?	Chronic pain	No/Yes
**Category 3: Eating habits and addictive-substances consumption**	Q13. Is your diet balanced and varied?	Healthy diet	No/Yes
	Q14. How many times a day do you eat?	Eating frequency	(open)
	Q15. Did you have breakfast today?	Breakfast	No/Yes
	Q16. Do you consume … Tobacco Alcohol Marijuana Energy drinks Drugs	Tobacco smoking Alcohol consumption Marijuana use Energy drinks consumption Drugs use	No/Sometimes/Yes
**Category 4: Use of technology and other habits**	Q17. How often do you check (open or unlock) your mobile phone per day?	Mobile phone use	Every less than 30 min/Between 30 and 60 min/Every more than 60 min/I don’t have a mobile phone
	Q18. When you study, do you keep your mobile phone notifications turned on?	Mobile notifications	No/Yes
	Q19. What do you do before bed?	Before-bed habits	Relaxing activities/Read/Homework/Mobile phone, computer/Stress-busting activities
	Q20. How many hours do you exercise a week?	Weekly exercise hours	(open)
	Q21. Are you a regular reader?	Regular reader	No/Yes
	Q22. What time of day do you work best?	Productive time of the day	Morning/Noon/Afternoon/Evening
**Category 5: Other personal conditions before and during the test**	Q23. How many hours did you sleep last night?	Hours of sleep	(open)
	Q24. How many hours have you been without drinking water?	Time without drinking water	Less than 1 h/From 1 h to 3 h/ More than 3 h
	Q25. Did you want to go to the toilet during the test?	Need to go to the toilet	No/Yes
	Q26. Do you have an interest in doing the test?	Interest in the experiment	No/Yes
	Q27. Do you have an exam today?	Exam	No/Yes
	Q28. Have you received some bad news recently?	Bad news received	No/Yes
	Q29. How many hours has it been since you received the bad news?	Hours bad news received	(open)
**Category 6:** **Perceived classroom conditions during the test**	Q30. Was there noise during the test?	Noise in classroom	No/Yes
	Q31. In the classroom, it is…	Temperature in classroom	Ok/Hot/Cold
	Q32. Does the classroom smell?	Smelly classroom	No/Yes
**Questions added by the researchers: Sociodemographic information**	Q33. Age	Age	2002/2003/2004/2005
	Q34. Gender	Gender	Female/Male/Other
	Q35. What is the highest level of school your mother has completed?	Maternal education level	University/Upper secondary education/Post-secondary vocational education/Lower secondary education/Didn’t finish primary school/I don’t know
	Q36. What is your mother’s main job?	Maternal occupational social class	(open)
	Q37. What does your mother do at her main job?	Maternal work task	(open)
	Q38. What is the highest level of school your father has completed?	Paternal education level	University/Upper secondary education/Post-secondary vocational education/Lower secondary education/Didn’t finish primary school/I don’t know
	Q39. What is your father’s main job?	Paternal occupational social class	(open)
	Q40. What does your father do at his main job?	Paternal work task	(open)
	Q41. Were you born in another country?	Country of birth	No/Yes
	Q42. Do you and your family have financial difficulties to afford basic needs?	Struggling to afford basic needs (poverty)	No/Yes

**Table 2:** Attentiveness (HRT-SE) according to participants’ sociodemographic characteristics (*n* = 1667).

Characteristics (% Missing)	N (%) or N (Median, IQR)	HRT-SE Median (IQR) or Spearman’s Test Correlation Coefficient	*p*-Value ^1^
**Gender (0%)**			
Female	839 (50.3)	166.8 (114.8; 245.1)	0.01 *
Male	822 (49.3)	151.0 (108.7; 230.6)	
Other	6 (0.4)	197.1 (130.1; 269.5)	
**Age (0%), years**	1667 (14.8, 14.6–14.9)	0.10	<0.01 *
Country of birth (0.2%)			
Spain	1421 (85.5)	153.24 (109.0; 234.1)	<0.01 *
Other	242 (14.5)	187.3 (128.8; 267.8)	
**PMA-R score (0.1%), number**	1665 (16, 12–20)	−0.39	<0.01 *
**Poverty—Struggling to afford basic needs**			
Yes	120 (7.2)	187.1 (141.3; 299.1)	<0.01 *
No	1540 (92.8)	155.4 (109.3; 234.8)	
**Maternal occupational social class (0%)**			
Highly skilled	607 (36.4)	138.6 (101.7; 213.0)	<0.01 *
Non-manual	552 (33.1)	161.6 (112.1; 241.2)	
Manual	79 (4.7)	181.5 (122.6; 288.7)	
Other	429 (25.7)	178.7 (127.0; 261.8)	
**Maternal education level (0.2%)**			
University	722 (43.4)	141.2 (102.4; 209.5)	<0.01 *
Upper secondary education	193 (11.6)	178.5 (118.3; 261.1)	
Post-secondary vocational education	177 (10.6)	169.4 (116.0; 262.4)	
Lower secondary education	218 (13.1)	183.0 (128.2; 286.5)	
Didn’t finish primary school	42 (2.5)	197.2 (146.4; 268.2)	
I don’t know/I don’t have a mother	311 (18.7)	164.9 (115.8; 248.3)	
**Paternal occupational social class (0%)**			
Highly skilled	511 (30.7)	143.7 (104.8; 213.9)	<0.01 *
Non-manual	487 (29.2)	150.9 (108.3; 232.0)	
Manual	336 (20.2)	180.7 (121.3; 262.0)	
Other	333 (20.0)	169.8 (120.3; 270.3)	
**Paternal education level (2%)**			
University	584 (35.7)	141.4 (102.0; 206.7)	<0.01 *
Upper secondary education	199 (12.2)	158.7 (118.3; 256.7)	
Post-secondary education	160 (9.8)	173.4 (115.1; 241.1)	
Lower secondary education	261 (16.0)	167.7 (116.0; 240.8)	
Didn’t finish primary school	47 (2.9)	231.8 (115.5; 323.5)	
I don’t know/I don’t have a father	383 (23.4)	164.0 (114.8; 258.1)	

Note: Some participants did not answer every question (e.g., country of birth). Hence, some sociodemographic variables do not total up to 1667. Maternal and paternal occupational social class were determined according to occupation/the job reported by the students and classified in accordance with the National Occupational Codes of 2011 (CNO-11). All variables were reported by the students. ^1^ *p*-value from Mann–Whitney U-test, Kruskal–Wallis or Spearman’s test. * statistical significance at the 0.05 level.

## Data Availability

The data presented in this study are openly available in Zenodo at https://doi.org/10.5281/zenodo.4896864 (accessed on 8 June 2021).

## Supplementary Materials

The following are available online at https://www.mdpi.com/article/10.3390/ijerph18158221/s1, Table S1: Crude associations between adolescent’s HRT-SE and factors included in the questionnaire (*n* = 1658), Table S2: Crude associations between secondary attention outcomes and factors included in the questionnaire (*n* = 1658), Table S3: Adjusted association between attentiveness (HRT-SE) and factors included in the questionnaire that showed a significant crude association (*n* = 1658), Table S4: Adjusted association between attentiveness (HRT-SE) and factors included in the questionnaire that showed a significant crude association (*n* = 1658) when using class group as strata, Table S5: Adjusted association between attentiveness (HRT-SE) and factors included in the questionnaire that showed a significant crude association (*n* = 1658) stratified by gender, Table S6: Adjusted association between attentiveness (HRT-SE) and factors included in the questionnaire that showed a significant crude association (*n* = 1658) stratified by maternal occupational social class, Table S7: Adjusted association between secondary attention outcomes and factors included in the questionnaire that showed a significant crude association (*n* = 1658)
