# Supplementary material for: Identifying Factors Influencing Attention in Adolescents with a Co-Created Questionnaire: A Citizen Science Approach with Secondary Students in Barcelona, Spain
Source: Int J Environ Res Public Health. 2021 Aug 3;18(15):8221. doi: 10.3390/ijerph18158221 (PMC8345951; doi:10.3390/ijerph18158221)
Supplement: Supplementary file 1 [file ijerph-18-08221-s001.zip › ijerph-1305348-supplementary.pdf]

SUPPLEMENTARY MATERIAL

Table S1. Crude associations between adolescent's HRT−SE and factors included in the questionnaire (n = 1,658)

Factors	Total n (%)	Change (%)[a], (95% CI)	p−value
		Category 1: Social relationships and interactions	
**Family relationship quality**			
Good	1104 (66.63)	Ref.	
Stable	456 (27.52)	3.24 (−2.21, 8.68)	0.24
Bad	97 (5.85)	0.31 (−9.776, 10.37)	0.95
**Classmates relationship quality**			
Good	1557 (94.02)	Ref.	
Not very good	84 (5.07)	15.78 (3.51, 28.04)	0.01*
Bad	15 (0.91)	21.37 (−8.40, 51.16)	0.16
**Bullying victimization**			
No	1638 (98.97)	Ref.	
Yes	17 (1.03)	24.20 (−4.47, 52.86)	0.10
**Peer and family conflict**			
No	1164 (70.29)	Ref.	
Yes	492 (29.71)	8.80 (3.27, 14.33)	< 0.01*
**Feelings of love**			
No	864 (52.24)	Ref.	
Yes, but it doesn't affect me	602 (36.40)	6.70 (1.34, 12.06)	0.01*
Yes, and it affects me	188 (11.37)	8.54 (0.28, 16.81)	0.04*
		Category 2: Psychological and physical health	
**Self−rated health**			
Good	1162 (70.30)	Ref.	
Regular	459 (27.77)	2.63 (−2.72, 7.98)	0.34
Bad	32 (1.94)	22.91 (2.07, 43.75)	0.03*
**Medication intake**			
No	1427 (86.38)	Ref.	
Yes	225 (13.62)	10.50 (3.00, 17.99)	0.01*
**Medication effect**			
No	189 (84.00)	Ref.	
Yes	36 (16.00)	8.98 (−10.37, 28.34)	0.36
**Mental or physical effort**			
No	1377 (83.25)	Ref.	
Yes	277 (16.75)	3.59 (−2.88, 10.06)	0.28
**Significant life event**			
No	1066 (64.45)	Ref.	
Yes	588 (35.55)	0.74 (−4.16, 5.64)	0.77
**Period pain**			
No	228 (13.85)	Ref.	
I am a boy	775 (47.08)	−0.29 (−7.39, 6.81)	0.94
Yes, but I don't have it today	535 (32.50)	10.91 (2.62, 19.20)	0.01*
Yes, and today I have it	108 (6.56)	7.68 (−4.20, 19.57)	0.21
**Chronic pain**			
No	1570 (95.21)	Ref.	
Yes	79 (4.79)	4.59 (−6.86, 16.04)	0.43
		Category 3 : Eating habits and addictive−substances consumption	
**Healthy diet**			
No	271 (16.41)	Ref.	
Yes	1380 (83.59)	−5.44 (−11.38, 0.49)	0.07

**Eating frequency**			
Less than 4 times	349 (21.13)	Ref.	
4 times or more	1303 (78.87)	−6.68 (−12.00, −1.37)	0.01*
**Breakfast**			
No	347 (20.94)	Ref.	
Yes	1310 (79.06)	−6.98 (−12.30, −1.67)	0.01*
**Tobacco smoking**			
No	1370 (84.36)	Ref.	
Sometimes	166 (10.22)	8.84 (0.45, 17.22)	0.04*
Yes	88 (5.42)	29.52 (16.17, 42.87)	< 0.01*
**Alcohol consumption**			
No	1182 (72.60)	Ref.	
Sometimes	393 (24.14)	7.12 (1.28, 12.96)	0.02*
Yes	53 (3.26)	36.91 (18.90, 54.91)	< 0.01*
**Marijuana use**			
No	1487 (92.42)	Ref.	
Sometimes	87 (5.41)	17.70 (5.55, 29.85)	< 0.01*
Yes	35 (2.18)	36.32 (14.50, 58.13)	< 0.01*
**Energy drinks consumption**			
No	740 (45.04)	Ref.	
Sometimes	569 (34.63)	6.84 (1.21, 12.47)	0.02*
Yes	334 (20.33)	9.60 (2.77, 16.43)	0.01
**Drugs use**			
No	1544 (96.08)	Ref.	
Sometimes	43 (2.68)	7.29 (−8.34, 22.91)	0.36
Yes	20 (1.24)	27.29 (0.31, 54.27)	0.05
Category 4: Use of technology and other habits			
**Mobile phone use**			
Every less than 30 m	639 (38.59)	Ref.	
Between 30 m−60 m	696 (42.03)	−11.69 (−16.25, −7.13)	< 0.01*
Every more than 60 m	307 (18.54)	−9.26 (−15.20, −3.32)	< 0.01*
I don’t have mobile	14 (0.85)	−16.20 (−37.53, 5.13)	0.14
**Mobile notification**			
No	602 (36.37)	Ref.	
Yes	1053 (63.63)	7.62 (2.42, 12.82)	< 0.01*
**Before−bed habits**			
Relaxing activities	181 (10.95)	Ref.	
Read	162 (9.80)	−4.58 (−14.35, 5.18)	0.36
Homework	72 (4.36)	1.26 (−12.09, 14.62)	0.85
Mobile phone, computer or TV	1214 (73.44)	6.67 (−1.37, 14.72)	0.10
Stress−busting activities	24 (1.45)	2.88 (−18.27, 24.02)	0.79
**Weekly exercise hours**			
Less than 7 h	1099 (67.01)	Ref.	
7 h or more	541 (32.99)	−8.16 (−12.72, −3.61)	< 0.01*
**Regular reader**			
No	1144 (69.33)	Ref.	
Yes	506 (30.67)	−9.41 (−13.96, −4.85)	< 0.01*
**Productive time of the day**			
Morning	480 (28.99)	Ref.	
Noon	383 (23.13)	−2.48 (−8.82, 3.85)	0.44
Afternoon	528 (31.88)	0.11 (−5.88, 6.10)	0.97
Evening	265 (16.00)	−1.91 (−9.03, 5.21)	0.54
Category 5: Other personal conditions before and during the test			
**Hours of sleep**			
Less than 8 h	940 (58.06)	Ref.	
Between 8 h and 10 h	669 (41.32)	−0.22 (−5.00, 4.57)	0.93
> 10 h	10 (0.62)	−0.69 (−30.63, 29.27)	0.97
**Time without drinking water**			
Less than 1	122 (7.36)	Ref.	
From 1 to 3	777 (46.86)	−9.21 (−17.58, −0.84)	0.03

More than 3	759 (45.78)	−9.82 (−18.15, −1.49)	0.02
**Need to go to the toilet**			
No	1406 (84.80)	Ref.	
Yes	252 (15.20)	7.64 (0.67, 14.61)	0.03*
**Interest in the experiment**			
No	166 (10.04)	Ref.	
Yes	1488 (89.96)	−12.55 (−19.32, −5.79)	< 0.01*
**Exam**			
No	1392 (83.96)	Ref.	
Yes	266 (16.04)	−5.06 (−11.08, 0.96)	0.10
**Bad news received**			
No	1166 (70.37)	Ref.	
Yes	491 (29.63)	8.33 (2.72, 13.72)	< 0.01*
**Hours bad news received**			
< 12 h	122 (25.79)	Ref.	
Between 12 h and 24 h	176 (37.21)	−6.89 (−17.57, 3.79)	0.21
> 24 h	175 (37.00)	−2.86 (−14.02, 8.30)	0.62
Category 6: Perceived classroom conditions during the test			
**Noise in classroom**			
No	817 (49.28)	Ref.	
Yes	841 (50.72)	−5.66 (−10.04, −1.27)	0.01*
**Temperature in classroom**			
OK	1136 (68.64)	Ref.	
Hot	199 (12.02)	−0.36 (−7.63, 6.90)	0.92
Cold	320 (19.34)	−0.01 (−6.02, 5.99)	1.00
**Smelly classroom**			
No	1531 (92.56)	Ref.	
Yes	123 (7.44)	1.23 (−7.76, 10.22)	0.79

Note: CI, confidence interval; HRT−SE, hit reaction time standard error (ms)

[a] Associations are estimated using linear regression and reported as percent change in the median of the outcome. * statistical significance at the 0.05 level.

Table S2. Crude associations between secondary attention outcomes and factors included in the questionnaire (n = 1,658)

	Impulsivity		Selective attention score		Alerting score		Orienting score		Executive attention	
**Factors**	**Change (count), (95% CI)**	**p−values**	**Change (count), (95% CI)**	**p−values**	**Change (ms), (95% CI)**	**p−value**	**Change (ms), (95% CI)**	**p−values**	**Change (ms), (95% CI)**	**p−values**
Category 1: Social relationships and interactions										
**Family relationship quality (ref. Good)**										
Stable	0.23 (−0.19,0.65)	0.28	0.09 (−0.07, 0.26)	0.28	2.51 (−3.66, 8.69)	0.42	1.62 (−4.38, 7.61)	0.59	1.10 (−4.15, 6.35)	0.68
Bad	1.05 (0.26, 1.85)	0.01*	0.28 (−0.03, 0.60)	0.08	−4.34 (−16.08, 7.40)	0.47	0.23 (−11.18, 11.64)	0.97	5.46 (−4.53, 15.44)	0.28
**Classmates relationship quality (ref. Good)**										
Not very good	0.40 (−0.46, 1.26)	0.36	0.23 (−0.10, 0.56)	0.18	−2.47 (−14.89, 9.95)	0.70	9.12 (−2.94, 21.18)	0.14	0.03 (−10.44, 10.50)	1.00
Bad	2.90 (0.92, 4.89)	0.01*	1.29 (0.52, 2.06)	< 0.01*	37.50 (8.73, 66.26)	0.01*	−15.03 (−42.96, 12.90)	0.29	58.70 (34.44, 82.95)	< 0.01*
**Bullying victimization (ref. No)**										
Yes	1.33 (−0.54, 3.20)	0.16	0.65 (−0.07, 1.37)	0.08	17.51 (−9.56, 44.57)	0.21	4.71 (−21.56, 30.97)	0.73	25.26 (2.35, 48.16)	0.03*
**Peer and family conflict (ref. No)**										
Yes	0.32 (−0.10, 0.73)	0.12	0.08 (−0.08, 0.24)	0.30	6.73 (0.77, 12.70)	0.03*	1.41 (−4.39, 7.20)	0.63	2.00 (−3.07, 7.08)	0.44
**Feelings of love (ref. No)**										
Yes, but it doesn’t affect me	0.45 (0.04, 0.86)	0.03*	0.05 (−0.11, 0.21)	0.54	7.61 (1.72, 13.50)	0.01*	−0.21 (−5.51, 5.93)	0.94	5.67 (0.67, 10.68)	0.03*
Yes, and it affects me	0.64 (0.02, 1.25)	0.04*	0.17 (−0.06, 0.41)	0.15	0.82 (−8.10, 9.75)	0.86	6.24 (−2.44, 14.91)	0.16	3.54 (−4.04, 11.13)	0.36
Category 2: Psychological and physical health										
**Self−rated health (ref. Good)**										
Regular	0.03 (−0.39, 0.45)	0.90	0.05 (−0.11, 0.21)	0.56	0.27 (−5.84, 6.39)	0.93	0.82 (−5.12, 6.76)	0.79	−1.37 (−6.56, 3.83)	0.61
Bad	−0.27 (−1.64, 1.10)	0.70	0.37 (−0.16, 0.90)	0.18	13.62 (−6.25, 33.50)	0.18	−6.62 (−25.92, 12.69)	0.50	6.36 (−10.54, 23.26)	0.46
**Medication intake (ref. No)**										
Yes	0.32 (−0.23, 0.88)	0.25	0.36 (0.15, 0.57)	< 0.01*	4.65 (−3.31, 12.60)	0.25	1.30 (−6.43, 9.03)	0.74	−0.58 (−7.34, 6.19)	0.87
**Medication effect (ref. No)**										
Yes	0.01 (−1.46, 1.47)	0.99	−0.11 (−0.86, 0.64)	0.78	−5.71 (−28.86, 17.43)	0.63	2.01 (−19.16, 23.17)	0.85	1.42 (−17.54, 20.39)	0.88
**Mental or physical effort (ref. No)**										
Yes	0.32 (−0.19, 0.82)	0.22	0.12 (−0.08, 0.32)	0.23	−2.74 (−10.05, 4.57)	0.46	2.07 (−5.02, 9.17)	0.57	3.26 (−2.95, 9.48)	0.30
**Significant life event (ref. No)**										
Yes	−0.22 (−0.60, 0.17)	0.27	0.03 (−0.11, 0.18)	0.64	2.02 (−3.66, 7.70)	0.49	−2.91 (−8.40, 2.59)	0.30	−0.50 (−5.33, 4.33)	0.84
**Period pain (ref. No)**										
I am a boy	1.12 (0.55, 1.69)	< 0.01*	−0.05 (−0.27, 0.17)	0.66	−1.87 (−10.25, 6.51)	0.66	−2.52 (−10.64, 5.61)	0.54	2.63 (−4.50, 9.75)	0.47
Yes, but I don’t have it today	−0.18 (−0.78, 0.42)	0.55	0.13 (−0.11, 0.36)	0.29	2.34 (−6.46, 11.13)	0.60	−1.42 (−9.94, 7.11)	0.75	0.88 (−6.60, 8.36)	0.82
Yes, and today I have it	−0.28 (−1.17, 0.61)	0.54	−0.07 (−0.42, 0.28)	0.70	5.28 (−7.71, 18.27)	0.43	−2.23 (−14.83, 10.36)	0.73	2.13 (−8.91, 13.18)	0.71
**Chronic pain (ref. No)**										
Yes	0.91 (0.03, 1.80)	0.05	0.12 (−0.23, 0.46)	0.50	−4.00 (−16.81, 8.80)	0.46	11.40 (−1.01, 23.81)	0.07	−5.92 (−16.81, 4.97)	0.29
Category 3 : Eating habits and addictive−substances consumption										
**Healthy diet (ref. No)**										
Yes	0.04 (−0.47, 0.55)	0.88	−0.36 (−0.55, 0.17)	< 0.01*	−4.44 (−11.81, 2.94)	0.24	−5.14 (−12.27, 1.99)	0.16	1.29 (−4.98, 7.55)	0.69
**Eating frequency (ref. Less than 4 times)**										
4 times or more	−0.48 (−0.94, −0.02)	0.04*	−0.11 (−0.29, 0.07)	0.21	−1.28 (−7.96, 5.41)	0.71	3.19 (−3.30, 9.68)	0.34	−3.62 (−9.29, 2.05)	0.21
**Breakfast (ref. No)**										
Yes	−0.49 (−0.95. 0.03)	0.04*	−0.10 (−0.28, 0.08)	0.28	−4.24 (−10.94, 2.47)	0.22	−4.57 (−11.07, 1.93)	0.17	−2.40 (−8.10, 3.29)	0.41
**Tobacco smoking (ref. No)**										
Sometimes	−0.35 (−0.97, 0.26)	0.26	0.03 (−0.21, 0.27)	0.80	5.70 (−3.43, 14.84)	0.22	−0.75 (−9.62, 8.12)	0.87	−2.85 (−10.35, 4.66)	0.46
Yes	0.72 (−0.10, 1.54)	0.09	0.68 (0.36, 1.01)	< 0.01*	−5.15 (−17.37, 7.07)	0.41	0.01 (−11.86, 11.88)	1.00	8.01 (−2.03, 18.04)	0.12
**Alcohol consumption (ref. No)**										
Sometimes	0.09 (−0.35, 0.52)	0.69	0.22 (0.05, 0.39)	0.01*	3.78 (−2.67, 10.24)	0.25	6.97 (0.69, 13.24)	0.03*	4.24 (−1.10, 9.58)	0.12
Yes	1.68 (0.63, 2.72)	< 0.01*	0.80 (0.39, 1.22)	< 0.01*	−3.22 (−18.78, 12.34)	0.69	13.10 (−2.03, 28.23)	0.09	14.02 (1.14, 26.90)	0.03*
**Marijuana use (ref. No)**										
Sometimes	0.88 (0.06, 1.70)	0.04*	0.45 (0.12, 0.77)	0.01*	7.70 (−4.55, 19.95)	0.22	11.80 (−0.11, 23.71)	0.05	3.97 (−6.02, 13.96)	0.44
Yes	2.16 (0.88, 3.43)	< 0.01*	1.18 (0.68, 1.69)	< 0.01*	−4.82 (−23.81, 14.17)	0.62	11.30 (−7.16, 29.76)	0.23	19.85 (4.37, 35.33)	0.01*
**Energy drinks consumption (ref. No)**										
Sometimes	0.48 (0.06, 0.89)	0.03*	0.16 (−0.01, 0.33)	0.06	3.55 (−2.63, 9.73)	0.26	−1.29 (−7.31, 4.72)	0.67	3.22 (−2.05, 8.49)	0.23
Yes	0.85 (0.36, 1.35)	< 0.01*	0.28 (0.08, 0.48)	0.01*	2.50 (−4.81, 9.81)	0.50	2.28 (−4.83, 9.39)	0.53	−0.23 (−6.46, 5.99)	0.94
**Drugs use (ref. No)**										
Sometimes	0.98 (−0.18, 2.14)	0.09	0.13 (−0.33, 0.59)	0.57	12.05 (−5.10, 29.20)	0.17	−9.22 (−25.94, 7.50)	0.28	8.10 (−6.01, 22.21)	0.26

Yes	0.68 (−1.01, 2.36)	0.43	0.08 (−0.59, 0.75)	0.81	32.99 (8.03, 57.96)	0.01*	5.39 (−18.95, 29.73)	0.66	2.63 (−17.91, 23.16)	0.80
Category 4: Use of technology and other habits										
**Mobile phone use (ref. Every 30 m or less)**										
Between 30 m−60 m	−0.68 (−1.10, −0.27)	< 0.01*	−0.29 (−0.45, −0.12)	< 0.01*	−2.24 (−8.33, 3.84)	0.47	−0.67 (−6.57, 5.24)	0.83	−4.80 (−9.97, 0.36)	0.07
Every more than 60 m	−0.38 (−0.91, 0.15)	0.16	−0.21 (−0.41, −0.00)	0.05	−1.30 (−9.02, 6.41)	0.74	−1.72 (−9.20, 5.77)	0.65	−6.70 (−13.24, −0.15)	0.05
I don't have mobile	−0.81 (−2.87, 1.25)	0.44	−0.26 (−1.06, 0.54)	0.52	14.94 (−15.06, 44.95)	0.33	11.94 (−17.18, 41.06)	0.42	3.50 (−21.97, 28.96)	0.79
**Mobile notification (ref. No)**										
Yes	−0.10 (−0.49, 0.30)	0.63	0.01 (−0.14, 0.16)	0.87	0.80 (−4.88, 6.48)	0.78	4.35 (−1.14, 9.85)	0.12	−0.19 (−5.01, 4.63)	0.94
**Before−bed habits (ref. Relaxing activities)**										
Read	−0.26 (−1.09, 0.57)	0.53	−0.24 (−0.56, 0.08)	0.14	−3.16 (−15.19, 8.86)	0.61	3.63 (−8.03, 15.29)	0.54	−1.63 (−11.84, 8.57)	0.75
Homework	−0.01 (−1.06, 1.08)	0.98	−0.21 (−0.62, 0.20)	0.32	−4.81 (−20.30, 10.69)	0.54	6.19 (−8.83, 21.21)	0.42	−2.42 (−15.57, 10.73)	0.72
Mobile phone, computer or TV	0.30 (−0.31, 0.91)	0.34	0.12 (−0.12, 0.35)	0.34	−2.53 (−11.39, 6.33)	0.58	4.53 (−4.06, 13.13)	0.30	5.05 (−2.47, 12.57)	0.19
Stress−busting activities	0.42 (−1.25, 2.08)	0.62	−0.36 (−1.01, 0.28)	0.27	−14.23 (−38.39, 9.92)	0.25	−2.92 (−26.34, 20.50)	0.81	7.17 (−13.33, 27.67)	0.49
**Weekly exercise hours (ref. < 7 h)**										
7 h or more	0.28 (−0.12, 0.68)	0.17	−0.19 (−0.35, −0.04)	0.02*	−4.76 (−10.57, 1.04)	0.11	3.12 (−2.54, 8.78)	0.28	−1.69 (−6.65, 3.27)	0.50
**Regular reader (ref. No)**										
Yes	−0.81 (−1.21, −0.40)	< 0.01*	−0.24 (−0.40, −0.08)	< 0.01*	−0.41 (−6.34, 5.52)	0.89	−2.69 (−8.43, 3.04)	0.36	−4.35 (−9.38, 0.68)	0.90
**Productive time of the day (ref. Morning)**										
Noon	0.16 (−0.37, 0.68)	0.56	0.09 (−0.11, 0.29)	0.39	2.94 (−4.65, 10.54)	0.43	−0.36 (−7.74, 7.01)	0.92	−2.03 (−8.49, 4.43)	0.54
Afternoon	−0.00 (−0.49, 0.48)	0.99	−0.02 (−0.21, 0.16)	0.80	1.88 (−5.11, 8.87)	0.60	1.12 (−5.67, 7.91)	0.75	−2.81 (−8.76, 3.13)	0.35
Evening	0.20 (−0.38, 0.79)	0.50	0.13 (−0.09, 0.36)	0.25	−2.39 (−10.87, 6.09)	0.58	3.39 (−4.86, 11.63)	0.42	−6.04 (−13.25, 1.18)	0.10
Category 5: Other personal conditions before and during the test										
**Hours of sleep (ref. < 8 h)**										
Between 8 hrs and 10 h	−0.24 (−0.63, 0.15)	0.23	−0.06 (−0.21, 0.09)	0.62	−1.98 (−7.61, 3.65)	0.49	0.35 (−5.06, 5.76)	0.90	−0.84 (−5.63, 3.95)	0.73
< 10 h	−1.14 (−3.59, 1.31)	0.36	−0.05 (−0.99, 0.89)	0.40	8.85 (−26.52, 44.22)	0.62	18.86 (−15.13, 52.86)	0.28	−4.41 (−34.52, 25.71)	0.77
**Time without drinking water (ref. Less than 1)**										
From 1 to 3	−0.42 (−1.16, 0.33)	0.27	−0.31 (−0.60, −0.02)	0.04*	−4.79 (−15.60, 6.02)	0.39	3.24 (−7.25, 13.73)	0.55	−9.11 (−18.29, 0.07)	0.05
More than 3	−0.51 (−1.26, 0.23)	0.18	−0.31 (−0.60, .0.02)	0.04*	−4.04 (−14.87, 6.79)	0.47	2.51 (−7.99, 13.02)	0.64	−8.43 (−17.62, 0.76)	0.07
**Need to go to the toilet (ref. No)**										
Yes	0.17 (−0.36, 0.69)	0.53	0.02 (−0.18, 0.23)	0.82	−3.00 (−10.59, 4.59)	0.44	1.38 (−5.98, 8.75)	0.71	0.45 (−6.01, 6.90)	0.89
**Interest in the experiment (ref. No)**										
Yes	−0.85 (−1.48, −0.23)	0.01*	−0.36 (−0.60, −0.12)	< 0.01*	1.83 (−10.92, 7.25)	0.69	−1.77 (−10.58, 7.05)	0.69	−0.88 (−8.60, 6.85)	0.82
**Exam (ref. No)**										
Yes	0.09 (−0.42, 0.60)	0.73	−0.20 (−0.40, −0.01)	0.04*	−5.93 (−13.36, 1.49)	0.12	−0.81 (−8.02, 6.40)	0.83	−6.92 (−13.22, −0.62)	0.03*
**Bad news received (ref. No)**										
Yes	0.35 (−0.06, 0.76)	0.09	0.26 (0.10, 0.41)	< 0.01*	0.99 (−4.97, 6.95)	0.74	0.81 (−4.98, 6.61)	0.78	0.60 (−4.47, 5.67)	0.82
**Hours bad news received (ref. < 12 h)**										
Between 12 h and 24 h	0.24 (−0.70, 1.18)	0.61	−0.61 (−1.05, −0.16)	0.01*	21.25 (7.07, 35.43)	< 0.01*	−9.65 (−23.79, 4.48)	0.18	5.76 (−5.12, 16.64)	0.30
> 24 h	−0.22 (−1.16, 0.72)	0.65	−0.64 (−1.09, −0.19)	0.01*	6.49 (−7.70, 20.69)	0.37	−14.37 (−28.52, −0.23)	0.05	1.08 (−9.81, 11.97)	0.85
Category 6: Perceived classroom conditions during the test										
**Noise in classroom (ref. No)**										
Yes	0.23 (−0.16, 0.60)	0.25	0.02 (−0.13, 0.16)	0.81	−4.26 (−9.71, 1.19)	0.13	0.30 (−4.99, 5.59)	0.91	−6.95 (−11.57, −2.33)	< 0.01*
**Temperature in classroom (ref. Ok)**										
Hot	0.38 (−0.21, 0.96)	0.21	−0.07 (−0.30, 0.16)	0.56	1.33 (−7.20, 9.85)	0.76	5.36 (−2.91, 13.63)	0.20	−2.11 (−9.36, 5.14)	0.57
Cold	0.06 (−0.42, 0.55)	0.80	0.03 (−0.16, 0.22)	0.83	6.08 (−0.94, 13.10)	0.09	4.88 (−1.93, 11.69)	0.16	−3.11 (−9.08, 2.86)	0.31
**Smelly classroom (ref. No)**										
Yes	0.91 (0.20, 1.64)	0.01*	0.10 (−0.18, 0.38)	0.47	6.09 (−4.31, 16.49)	0.25	−7.55 (−17.64, 2.54)	0.14	0.29 (−8.56, 9.13)	0.95

Associations are estimated using linear regression and reported as additive change in the mean of outcome. * statistical significance at the 0.05 level.

Table S3. Adjusted association between attentiveness (HRT−SE) and factors included in the questionnaire that showed a significant crude association (n = 1,658)

Variables	Change (%)	95% CI	p−values
Category 1: Social relationships and interactions			
**Classmates relationship quality** (ref. Good)			
Not very good	14.33	(3.37, 25.29)	0.01*
Bad	0.74	(−21.77, 23.25)	0.95
**Peer and family conflict** (ref. No)			
Yes	5.61	(0.67, 10.52)	0.03*
**Feelings of love** (ref. No)			
Yes, but it doesn't affect me	3.25	(−1.44, 7.94)	0.17
Yes, and it affects me	2.01	(−5.02, 9.04)	0.58
Category 2: Psychological and physical health			
**Self−rated health** (ref. Good)			
Regular	3.38	(−1.52, 8.29)	0.18
Bad	16.32	(−1.41, 34.05)	0.07
**Medication intake** (ref. No)			
Yes	9.98	(3.26, 16.69)	< 0.01*
**Period pain** (ref. No)			
I am a boy	0.10	(−14.44, 14.63)	0.99
Yes, but I don't have it today	8.12	(0.67, 15.57)	0.03*
Yes, and today I have it	5.08	(−5.38, 15.53)	0.34
Category 3 : Eating habits and addictive−substances consumption			
**Eating frequency** (ref. < 4 times)			
4 times or more	−0.54	(−5.75, 4.68)	0.84
**Breakfast** (ref. No)			
Yes	−2.78	(−7.85, 2.29)	0.28
**Tobacco smoking** (ref. No)			
Sometimes	5.74	(−1.70, 13.18)	0.13
Yes	16.19	(5.07, 27.32)	< 0.01*
**Alcohol consumption** (ref. No)			
Sometimes	3.40	(−1.79, 8.58)	0.20
Yes	17.22	(2.98, 31.46)	0.02*
**Marijuana use** (ref. No)			
Sometimes	10.09	(−0.26, 20.44)	0.06
Yes	21.54	(3.76, 39.32)	0.02*
**Energy drinks consumption** (ref. No)			
Sometimes	4.06	(−0.97, 9.09)	0.11
Yes	4.95	(−1.16, 11.07)	0.11
Category 4: Use of technology and other habits			
**Mobile phone use** (ref. Every 30 m or less)			
Between 30 m−60 m	−5.34	(−9.82, −0.87)	0.02*
Every more than 60 m	−5.30	(−10.92, 0.32)	0.07
I don't have a mobile	−22.94	(−40.86, −5.01)	0.01*
**Mobile notification** (ref. No)			
Yes	1.74	(−2.75, 6.23)	0.45
**Weekly exercise hours** (ref. < 7 h)			
7 h or more	−3.61	(−8.05, 0.83)	0.11
**Regular reader** (ref. No)			
Yes	−5.80	(−10.29, −1.31)	0.01*
Category 5: Other personal conditions before and during the test			
**Need to go to the toilet** (ref. No)			
Yes	4.78	(−1.40, 10.95)	0.13
**Interest in the experiment** (ref. No)			
Yes	−7.36	(−13.91, −0.82)	0.03*
**Bad news received** (ref. No)			
Yes	7.93	(2.94, 12.92)	< 0.01*
Category 6: Perceived classroom conditions during the test			
**Noise in classroom** (ref. No)			
Yes	−1.18	(−5.37, 3.02)	0.58
Factors added by the researchers (Socio−demographic characteristics)			
**Gender** (ref. Female)			
Male	−12.02	(−15.77, −8.27)	< 0.01*
Other	6.13	(−31.00, 43.27)	0.75
**Age**	7.66	(1.06, 14.26)	0.02*
**Country of birth** (ref. Spain)			
Other	−0.49	(−6.69, 5.71)	0.88
**PMA−R score**	−3.05	(−3.41, −2.69)	< 0.01*
**Maternal occupational social class** (ref. Highly skilled)			

Non−manual	5.84	(0.42, 11.26)	0.04*
Manual	11.42	(−0.19, 23.04)	0.05
Other	6.31	(0.10, 12.52)	0.05
**Struggling to afford basic needs (poverty)** (ref. No)			
Yes	13.37	(4.03, 22.71)	0.01*

Note: CI, confidence interval; HRT−SE, hit reaction time standard error. Model adjusted for: sex, age, country of birth, PMA−R, maternal occupational social class and poverty. Associations are estimated using conditional linear regression with school as strata. Association reported as percent change in the median of the outcome. * statistical significance at the 0.05 level.

Table S4. Adjusted association between attentiveness (HRT−SE) and factors included in the questionnaire that showed a significant crude association (n = 1,658) when using class group as strata.

Variables	Change (%)	95% CI	p−values
Category 1: Social relationships and interactions			
**Classmates relationship quality** (ref. Good)			
Not very good	14.80	(3.90, 25.71)	0.01*
Bad	0.65	(−21.64, 22.95)	0.95
**Peer and family conflict** (ref. No)			
Yes	5.44	(0.54, 10.33)	0.03*
**Feelings of love** (ref. No)			
Yes, but it doesn't affect me	3.65	(−1.01, 8.31)	0.13
Yes, and it affects me	1.94	(−5.03, 8.91)	0.59
Category 2: Psychological and physical health			
**Self−rated health** (ref. Good)			
Regular	3.15	(−1.72, 8.02)	0.20
Bad	17.76	(−0.04, 35.57)	0.05
**Medication intake** (ref. No)			
Yes	9.84	(3.20, 16.48)	< 0.01*
**Period pain** (ref. No)			
I am a boy	0.23	(−14.22, 14.68)	0.98
Yes, but I don't have it today	8.14	(0.76, 15.52)	0.03*
Yes, and today I have it	4.07	(−6.19, 14.32)	0.44
Category 3 : Eating habits and addictive−substances consumption			
**Eating frequency** (ref. < 4 times)			
4 times or more	−0.00	(−0.06, 0.05)	0.89
**Breakfast** (ref. No)			
Yes	−2.63	(−7.74, 2.49)	0.31
**Tobacco smoking** (ref. No)			
Sometimes	5.38	(−2.01, 12.77)	0.15
Yes	15.89	(4.91, 26.86)	0.01*
**Alcohol consumption** (ref. No)			
Sometimes	3.52	(−1.67, 8.70)	0.18
Yes	17.88	(3.70, 32.06)	0.01*
**Marijuana use** (ref. No)			
Sometimes	9.44	(−0.81, 19.70)	0.07
Yes	21.03	(3.47, 38.60)	0.02*
**Energy drinks consumption** (ref. No)			
Sometimes	4.19	(−0.82, 9.20)	0.10
Yes	4.92	(−1.16, 10.99)	0.11
Category 4: Use of technology and other habits			
**Mobile phone use** (ref. Every 30 m or less)			
Between 30 m−60 m	−5.60	(−10.04, −1.16)	0.01*
Every more than 60m	−5.31	(−10.90, 0.27)	0.06
I don't have mobile	−21.05	(−39.29, −2.80)	0.02*
**Mobile notification** (ref. No)			
No (ref.)			
Yes	2.16	(−2.30, 6.63)	0.34
**Weekly exercise hours** (ref. < 7 h)			
7 h or more	−3.65	(−8.10, 0.81)	0.11
**Regular reader** (ref. No)			
No (ref.)			
Yes	−5.84	(−10.29, −1.38)	0.01*
Category 5: Other personal conditions before and during the test			
**Need to go to the toilet** (ref. No)			
Yes	4.86	(−1.28, 11.00)	0.12
**Interest in the experiment** (ref. No)			
Yes	−7.62	(−14.09, −1.15)	0.02*
**Bad news received** (ref. No)			
Yes	8.04	(3.06, 13.01)	< 0.01*
Category 6: Perceived classroom conditions during the test			
**Noise in classroom** (ref. No)			
Yes	−1.13	(−5.36, 3.06)	0.60

Note: CI, confidence interval; HRT−SE, hit reaction time standard error. Model adjusted for: sex, age, country of birth, PMA−R, maternal occupational social class and poverty. Effects are calculated using conditional linear regression with class group as random effect. Association reported as percent change in the median of the outcome. * statistical significance at the 0.05 level.

Table S5. Adjusted association between attentiveness (HRT−SE) and factors included in the questionnaire that showed a significant crude association (n = 1,658) stratified by gender

Variables	FEMALE Change (%) 95% CI	p−value	MALE Change (%) 95% CI	p−value	P for interaction[a]
Category 1: Social relationships and interactions					
**Classmates relationship quality** (ref. Good)					
Not very good	17.43 (2.24, 32.63)	0.03*	10.38 (−5.61, 26.37)	0.20	0.77
Bad	−6.13 (−30.42, 18.16)	0.62	9.83 (−38.29, 57.96)	0.69	
**Peer and family conflict** (ref. No)					
Yes	7.47 (0.94, 13.99)	0.03*	3.95 (−3.73, 11.61)	0.31	0.64
**Feelings of love** (ref. No)					
Yes, but it doesn't affect me	4.31 (−2.42, 11.04)	0.21	2.83 (−3.82, 9.48)	0.40	0.94
Yes, and it affects me	2.39 (−7.00, 11.79)	0.62	1.37 (−9.38, 12.13)	0.80	
Category 2: Psychological and physical health					
**Self−rated health** (ref. Good)					
Regular	−0.08 (−6.26, 6.10)	0.98	7.62 (−0.37, 15.60)	0.06	0.21
Bad	13.44 (−10.05, 37.38)	0.26	18.80 (−7.94, 45.55)	0.17	
**Medication intake** (ref. No)					
Yes	6.45 (−2.16, 15.05)	0.14	17.05 (6.06, 28.04)	< 0.01*	0.11
**Period pain** (ref. No)					
I am a boy	Not applicable		Not applicable		
Yes, but I don't have it today	7.43 (0.06, 14.92)	0.05			
Yes, and today I have it	2.63 (−7.93, 13.19)	0.63			
Category 3 : Eating habits and addictive−substances consumption					
**Eating frequency** (ref. < 4 times)					
4 times or more	−0.03 (−0.11, 0.04)	0.39	0.01 (−0.07, 0.08)	0.89	0.20
**Breakfast** (ref. No)					
Yes	−5.44 (−12.43, 1.55)	0.13	0.04 (−7.42, 7.49)	0.99	0.22
**Tobacco smoking** (ref. No)					
Sometimes	4.84 (−4.66, 14.35)	0.32	7.60 (−4.43, 19.62)	0.13	0.32
Yes	10.68 (−2.95, 24.31)	0.12	24.70 (5.90, 43.49)	0.01*	
**Alcohol consumption** (ref. No)					
Sometimes	1.31 (−5.44, 8.06)	0.70	7.55 (−0.65, 15.76)	0.20	0.31
Yes	19.09 (0.67, 37.51)	0.04*	17.98 (−4.96, 40.92)	0.02*	
**Marijuana use** (ref. No)					
Sometimes	5.86 (−6.85, 18.57)	0.37	15.51 (−2.19, 33.22)	0.09	0.30
Yes	39.84 (7.96, 71.72)	0.01*	14.31 (−7.58, 36.19)	0.20	
**Energy drinks consumption** (ref. No)					
Sometimes	5.94 (−1.06, 12.94)	0.10	1.60 (−5.73, 8−93)	0.67	0.54
Yes	7.80 (−1.90, 17.50)	0.12	2.84 (−5.31, 10.99)	0.50	
Category 4: Use of technology and other habits					
**Mobile phone use** (ref. Every 30 m or less)					
Between 30 m−60 m	−6.07 (−12.17, 0.03)	0.05	−4.20 (−10.81, 2.43)	0.22	0.82
Every more than 60 m	−0.90 (−9.41, 7.61)	0.84	−8.64 (−16.27, −1.00)	0.03*	0.39
I don't have mobile	−18.67 (−43.27, 5.93)	0.12	−25.52 (−52.60, 1.57)	0.07	
**Mobile notification** (ref. No)					
Yes	−0.96 (−7.15, 5.24)	0.76	5.07 (−1.51, 11.65)	0.13	0.15
**Weekly exercise hours** (ref. < 7 h)					
7 h or more	−0.03 (−0.10, 0.04)	0.33	−0.03 (−0.09, 0.03)	0.38	
**Regular reader** (ref. No)					
Yes	−7.55 (−13.10, −1.99)	0.01*	−2.94 (−10.58, 4.70)	0.45	0.24
Category 5: Other personal conditions before and during the test					
**Need to go to the toilet** (ref. No)					
Yes	6.60 (−2.05, 15.25)	0.14	0.77 (−7.96, 9.50)	0.86	0.34
**Interest in the experiment** (ref. No)					
Yes	−10.66 (−20.42, −0.89)	0.03*	−5.05 (−13.99, 3.88)	0.27	0.28
**Bad news received** (ref. No)					
Yes	9.26 (2.53, 15.99)	0.01*	6.86 (−0.68, 14.40)	0.08	0.79
Category 6: Perceived classroom conditions during the test					
**Noise in classroom** (ref. No)					
Yes	−2.00 (−7.78, 3.77)	0.50	−0.02 (−6.14, 6.09)	0.99	0.40

Note: CI, confidence interval; HRT−SE, hit reaction time standard error. Model adjusted for: age, country of birth, PMA−R, maternal occupational social class and poverty. Effects are calculated using conditional linear regression with school as random effect. Association reported as percent change in the median of the outcome.

[a] Excluding the participants having selected "Other" in gender group

* statistical significance at the 0.05 level.

Table S6. Adjusted association between attentiveness (HRT−SE) and factors included in the questionnaire that showed a significant crude association (n = 1,658) stratified by maternal occupational social class.

Factors	Maternal occupational social class								
	Highly skilled Change (%) 95% CI	p−value	Non−manual Change (%) 95% CI	p−value	Manual Change (%) 95% CI	p−value	Other Change (%) 95% CI	p−value	P for interaction
Category 1: Social relationships and interactions									
**Classmates relationship quality** (ref. Good)									
Not very good	−0.10 (−18.28, 18.08)	0.99	21.18 (1.88, 40.48)	0.03*	40.80 (−39.36, 120.96)	0.32	23.72 (3.24, 44.20)	0.02*	0.18
Bad	−31.52 (−74.21, 11.17)	0.15	−3.32 (−51.66, 45.02)	0.89	−46.36 (−90.77, −1.96)	0.04*	14.31 (−18.09, 46.71)	0.39	
**Peer and family conflict** (ref. No)									
No (ref.)									
Yes	6.14 (−2.42, 14.71)	0.16	3.34 (−5.11, 11.79)	0.44	2.68 (−18.11, 23.46)	0.80	9.24 (−0.32, 18.81)	0.06	0.78
**Feelings of love** (ref. No)									
Yes, but it doesn't affect me	14.23 (5.59, 22.88)	< 0.01*	−4.23 (−11.69, 3.20)	0.26	20.05 (−5.00, 45.10)	0.12	0.05 (−9.03, 9.12)	0.99	0.05
Yes, and it affects me	1.70 (−10.90, 14.29)	0.79	−1.29 (−13.09, 10.51)	0.83	18.23 (−18.98, 55.44)	0.34	1.79 (−10.90, 14.47)	0.78	
Category 2: Psychological and physical health									
**Self−rated health** (ref. Good)									
Regular	−0.92 (−8.73, 6.89)	0.82	1.81 (−6.48, 10.11)	0.67	28.98 (−0.89, 58.85)	0.06	6.05 (−4.08, 16.17)	0.24	0.13
Bad	21.23 (−18.83, 61.27)	0.30	38.02 (3.81, 72.24)	0.03*	−5.05 (−63.48, 53.38)	0.87	−0.92 (−26.52, 24.67)	0.94	
**Medication intake** (ref. No)									
Yes	14.86 (3.43, 26.29)	0.01*	5.18 (−6.04, 16.39)	0.37	0.40 (−30.68, 31.48)	0.98	10.20 (−3.28, 23.68)	0.14	0.64
**Period pain** (ref. No)									
I am a boy	−4.41 (−31.73, 22.91)	0.75	7.36 (−20.08, 34.80)	0.60	−5.60 (−31.23, 20.02)	0.67	−6.61 (−28.78, 15.57)	0.56	0.17
Yes, but I don't have it today	8.31 (−4.28, 20.90)	0.20	9.42 (−3.15, 22.00)	0.14	30.03 (−6.12, 66.18)	0.10	−1.93 (−16.45, 12.59)	0.79	
Yes, and today I have it	10.36 (−8.76, 29.47)	0.29	−1.12 (−17.22, 14.98)	0.89	50.55 (−19.38, 120.49)	0.16	−4.75 (−24.13, 14.63)	0.63	
Category 3 : Eating habits and addictive−substances consumption									
**Eating frequency** (ref. < 4 times)									
4 times or more	−0.06 (−0.16, 0.03)	0.21	0.07 (−0.03, 0.16)	0.16	0.10 (−0.12, 0.31)	0.38	−0.04 (−0.13, 0.05)	0.37	0.15
**Breakfast** (ref. No)									
Yes	−5.64 (−14.83, 3.55)	0.23	−3.96 (−12.61, 4.68)	0.37	7.85 (−16.70, 32.40)	0.53	2.27 (−7.01, 11.54)	0.63	0.73
**Tobacco smoking** (ref. No)									
Sometimes	9.75 (−2.48, 21.97)	0.12	−1.51 (−13.46, 10.45)	0.81	1.13 (−32.33, 34.60)	0.95	9.81 (−6.93, 26.45)	0.25	0.37
Yes	11.36 (−6.44, 29.16)	0.21	7.79 (−11.05, 26.63)	0.42	−0.36 (−43.88, 43.16)	0.99	41.57 (17.73, 65.42)	< 0.01*	
**Alcohol consumption** (ref. No)									
Sometimes	3.41 (−5.14 ,11.95)	0.44	8.31 (−0.99, 17.62)	0.08	7.38 (−16.52, 31.27)	0.55	−3.53 (−13.45, 6.38)	0.49	0.49
Yes	13.05 (−9.72, 35.82)	0.26	21.48 (−9.02, 51.99)	0.17	−13.21 (−47.89, 21.48)	0.46	32.94 (4.82, 61.06)	0.02*	
**Marijuana use** (ref. No)									
Sometimes	15.94 (−4.89, 36.77)	0.13	5.39 (−9.88, 20.67)	0.49	39.92 (−19.86, 99.70)	0.19	10.28 (−9.91, 30.48)	0.32	0.17
Yes	19.75 (−13.53, 53.04)	0.25	0.44 (−24.75, 25.63)	0.97	−33.78 (−81.77, 14.22)	0.17	60.81 (22.17, 99.44)	< 0.01*	
**Energy drinks consumption** (ref. No)									
Sometimes	0.51 (−7.56, 8.58)	0.90	5.69 (−3.07, 14.45)	0.20	22.13 (−3.81, 48.07)	0.10	4.17 (−5.91, 14.25)	0.42	0.85
Yes	−1.02 (−11.31, 9.27)	0.85	11.82 (0.66, 22.97)	0.04*	21.71 (−6.71, 50.13)	0.13	3.47 (−7.76, 14.69)	0.55	
Category 4: Use of technology and other habits									
**Mobile phone use** (ref. Every 30 m or less)									
Between 30 m−60 m	−5.19 (−12.66, 2.28)	0.17	−3.67 (−11.50, 4.15)	0.36	−13.13 (−32.14, 5.88)	0.18	−6.19 (−15.12, 2.74)	0.17	0.88
Every more than 60 m	−6.31 (−16.09, 3.47)	0.21	−7.50 (−17.47, 2.47	0.14	−1.98 (−27.30, 23.34)	0.88	−2.03 (−12.45, 8.39)	0.70	
I don't have mobile	−29.18 (−57.52, −0.84)	0.04*	2.27 (−50.19, 54.72)	0.93	0	0	−23.37 (−50.10, 3.37)	0.09	
**Mobile notification** (ref. No)									
Yes	3.15 (−4.36, 10.67)	0.41	0.29 (−7.52, 8.10)	0.94	12.12 (−9.93, 34.17)	0.28	−0.47 (−9.03, 8.09)	0.92	0.42

**Weekly exercise hours** (ref. < 7 h)									
> 7 h	0.03 (−0.04, 0.11)	0.37	−0.06 (−0.14, 0.03)	0.19	0.13 (−0.10, 0.36)	0.28	−0.12 (−0.21, −0.03)	0.01*	0.09
**Regular reader** (ref. No)									
Yes	−3.84 (−11.29, 3.60)	0.31	−8.27 (−15.85, −0.69)	0.03*	−11.24 (−32.48, 10.00)	0.30	−2.81 (−12.21, 6.59)	0.56	0.76
Category 5: Other personal conditions before and during the test									
**Hours of sleep** (ref. < 6 h)									
Between 6 h and 7 h	4.81 (−9.68, 19.31)	0.52	−3.43 (−17.26, 10.40)	0.63	−12.58 (−42.81, 17.65)	0.42	−12.89 (−23.87, −1.91)	0.02*	0.44
> 7 h	2.14 (−12.13, 16.42)	0.77	3.98 (−11.20, 19.16)	0.61	−9.89 (−40.51, 20.73)	0.53	−10.22 (−21.72, 1.29)	0.08	
**Need to go to the toilet** (ref. No)									
Yes	−3.40 (−13.53, 6.73)	0.51	11.00 (−0.34, 22.33)	0.06	−5.77 (−29.84, 18.31)	0.64	7.50 (−3.90, 18.90)	0.20	0.30
**Interest in the experiment** (ref. No)									
Yes	1.72 (−11.92, 15.37)	0.81	−6.86 (−18.07, 4.36)	0.23	−27.68 (−47.03, −8.33)	0.01*	−13.05 (−23.82, −2.28)	0.02*	0.22
**Bad news received** (ref. No)									
Yes	13.02 (4.42, 21.63)	< 0.01*	6.84 (−1.78, 15.45)	0.12	3.11 (−15.96, 23.19)	0.76	5.41 (−4.32, 15.14)	0.28	0.75
Category 6: Perceived classroom conditions during the test									
**Noise in classroom** (ref. No)									
Yes	−2.00 (−8.98, 4.98)	0.57	1.26 (−6.33, 8.86)	0.74	−1.49 (−20.47, 17.50)	0.88	−3.12 (−11.01, 4.77)	0.44	0.84

Note: CI, confidence interval; HRT−SE, hit reaction time standard error. Model adjusted for: age, sex, country of birth, PMA−R and poverty. Effects are calculated using conditional linear regression with school as random effect. Association reported as percent change in the median of the outcome. * statistical significance at the 0.05 level.

Table S7. Adjusted association between secondary attention outcomes and factors included in the questionnaire that showed a significant crude association (n = 1,658)

Variables	Impulsivity		Selective attention score		Alerting score		Orienting score		Executive attention	
	Change (count), 95% (CI)	p−value	Change (count), 95% (CI)	p−value	Change (ms), 95% (CI)	p−value	Change (ms), 95% (CI)	p−value	Change (ms), 95% (CI)	p−value
Category 1: Social relationships and interactions										
**Classmates relationship quality** (ref. Good)										
Not very good	0.41 (−0.43, 1.24)	0.34	0.22 (−0.10, 0.54)	0.18	−3.38 (−15.84, 9.08)	0.60	9.00 (−3.12, 21.12)	0.15	−0.29 (−10.58, 10.01)	0.96
Bad	2.73 (0.79, 4.68)	0.01*	0.93 (0.18, 1.68)	0.02*	34.41 (5.39, 63.44)	0.02*	−15.56 (−43.81, 12.69)	0.28	51.65 (27.71, 75.60)	< 0.01*
**Peer and family conflict** (ref. No)										
Yes	0.49 (0.08, 0.90)	0.02*	0.03 (−0.13, 0.19)	0.71	6.24 (0.16, 12.32)	0.04*	1.25 (−4.67, 7.17)	0.68	1.86 (−3.20, 6.92)	0.47
**Feelings of love** (ref. No)										
Yes, but it doesn't affect me	0.27 (−0.12, 0.66)	0.18	−0.03 (−0.18, 0.12)	0.68	6.97 (1.07, 12.87)	0.02*	−0.43 (−6.18, 5.32)	0.88	3.83 (−1.09, 8.75)	0.13
Yes, and it affects me	0.53 (−0.07, 1.13)	0.09	0.05 (−0.18, 0.28)	0.69	−0.84 (−9.80, 8.11)	0.85	6.01 (−2.72, 14.74)	0.18	1.37 (−6.10, 8.84)	0.72
Category 2: Psychological and physical health										
**Self−rated health** (ref. Good)										
Regular	0.28 (−0.13, 0.69)	0.19	0.07 (−0.09, 0.23)	0.39	0.43 (−5.73, 6.60)	0.89	0.65 (−5.36, 6.65)	0.83	−0.12 (−5.26, 5.02)	0.96
Bad	−0.23 (−1.56, 1.10)	0.73	0.26 (−0.25, 0.78)	0.31	13.18 (−6.63, 32.98)	0.19	−7.07 (−26.37, 12.22)	0.47	4.03 (−12.50, 20.56)	0.63
**Medication intake** (ref. No)										
Yes	0.46 (−0.07, 0.99)	0.09	0.35 (0.14, 0.55)	< 0.01*	4.40 (−3.56, 12.35)	0.28	0.88 (−6.87, 8.63)	0.82	−0.36 (−7.00, 6.27)	0.91
**Period pain** (ref. No)										
I am a boy	0.70 (−0.57, 1.96)	0.28	0.09 (−0.40, 0.57)	0.73	−3.84 (−22.77, 15.09)	0.69	−18.05 (−36.43, 0.33)	0.05	−2.12 (−17.89, 13.66)	0.79
Yes, but I don't have it today	−0.24 (−0.85, 0.36)	0.43	0.08 (−0.15, 0.31)	0.51	1.93 (−7.05, 10.90)	0.67	0.24 (−8.47, 8.96)	0.96	0.60 (−6.88, 8.08)	0.88
Yes, and today I have it	−0.43 (−1.30, 0.43)	0.33	−0.11 (−0.44, 0.23)	0.53	4.58 (−8.38, 17.55)	0.49	−1.78 (−14.36, 10.81)	0.78	1.14 (−9.67, 11.94)	0.84
Category 3 : Eating habits and addictive−substances consumption										
**Eating frequency** (ref. < 4 times)										
4 times or more	−0.31 (−0.77, 0.14)	0.18	−0.21 (−0.36, −0.07)	< 0.01*	0.30 (−6.51, 7.10)	0.93	3.51 (−3.11, 10.13)	0.30	−0.21 (−5.86, 5.44)	0.94
**Breakfast** (ref. No)										
Yes	−0.37 (−0.82, 0.08)	0.11	−0.02 (−0.19, 0.16)	0.83	−2.94 (−0.71, 3.84)	0.40	−4.55 (−11.13, 2.03)	0.18	0.08 (−5.55, 5.71)	0.98
**Tobacco smoking** (ref. No)										
Sometimes	−0.26 (−0.86, 0.33)	0.39	−0.03 (−0.26, 0.21)	0.84	5.44 (−3.76, 14.63)	0.25	−1.53 (−10.49, 7.42)	0.74	−4.33 (−11.73, 3.07)	0.25
Yes	0.64 (−0.17, 1.46)	0.12	0.47 (0.15, 0.79)	< 0.01*	−7.24 (−19.69, 5.21)	0.26	−0.96 (−13.08, 11.17)	0.88	3.64 (−6.33, 13.61)	0.47
**Alcohol consumption** (ref. No)										
Sometimes	0.12 (−0.30, 0.55)	0.57	0.16 (−0.01, 0.32)	0.07	3.02 (−3.49, 9.54)	0.36	6.42 (0.07, 12.78)	0.05	3.25 (−2.04, 8.55)	0.23
Yes	1.56 (0.53, 2.59)	< 0.01*	0.52 (0.11, 0.92)	0.01*	−6.29 (−22.07, 9.50)	0.44	13.48 (−1.90, 28.86)	0.09	8.35 (−4.45, 21.16)	0.20
**Marijuana use** (ref. No)										
Sometimes	0.89 (0.08, 1.69)	0.03*	0.32 (0.00, 0.64)	0.05	5.77 (−6.54, 18.08)	0.36	10.79 (−1.21, 22.78)	0.08	0.94 (−8.90, 10.78)	0.85
Yes	1.84 (0.59, 3.09)	< 0.01*	0.95 (0.46, 1.46)	< 0.01*	−6.32 (−25.44, 12.79)	0.52	10.42 (−8.23, 29.08)	0.27	14.84 (−0.42, 30.10)	0.06
**Energy drinks consumption** (ref. No)										
Sometimes	0.13 (−0.29, 0.54)	0.55	0.10 (−0.06, 0.26)	0.23	3.11 (−3.16, 9.39)	0.33	−1.50 (−7.62, 4.63)	0.63	0.69 (−4.54, 5.93)	0.80
Yes	0.34 (−0.16, 0.83)	0.18	0.19 (−0.01, 0.38)	0.06	2.21 (−5.36, 9.77)	0.57	2.13 (−5.25, 9.51)	0.57	−4.87 (−11.18, 1.43)	0.13
Category 4: Use of technology and other habits										
**Mobile phone use** (ref. Every 30 m or less)										
Between 30 m−60 m	−0.55 (−0.96, −0.14)	0.01*	−0.15 (−0.31, 0.01)	0.06	−0.47 (−6.63, 5.69)	0.88	−0.81 (−6.80, 5.19)	0.79	−1.64 (−6.77, 3.48)	0.53
Every more than 60m	−0.46 (−0.97, 0.06)	0.08	−0.13 (−0.33, 0.07)	0.21	−0.12 (−7.87, 7.62)	0.98	−1.19 (−8.73, 6.35)	0.76	−4.63 (−11.08, 1.82)	0.16
I don't have mobile	−0.88 (−2.90, 1.14)	0.39	−0.44 (−1.22, 0.34)	0.27	11.15 (−19.15, 41.44)	0.47	10.15 (−19.33, 39.63)	0.50	1.80 (−23.38, 26.98)	0.89
**Mobile notification** (ref. No)										
Yes	−0.16 (−0.55, 0.22)	0.41	−0.09 (−0.24, 0.06)	0.22	−0.67 (−6.40, 5.07)	0.82	4.29 (−1.28, 9.85)	0.13	−2.40 (−7.16, 2.35)	0.32
**Weekly exercise hours** (ref. < 7 h)										
7 h or more	0.11 (−0.29, 0.51)	0.59	−0.10 (−0.26, 0.05)	0.19	−3.35 (−9.31, 2.60)	0.21	3.37 (−2.45, 9.19)	0.26	−0.52 (−5.50, 4.46)	0.84
**Regular reader** (ref. No)										
Yes	−0.25 (−0.67, 0.16)	0.23	−0.15 (−0.31, 0.01)	0.06	−0.06 (−6.27, 6.15)	0.98	−2.53 (−8.55, 3.49)	0.41	−0.43 (−5.58, 4.73)	0.87
Category 5: Other personal conditions before and during the test										
**Need to go to the toilet** (ref. No)										
Yes	0.11 (−0.40, 0.63)	0.67	−0.03 (−0.23, 0.17)	0.77	−4.09 (−11.74, 3.55)	0.29	1.16 (−6.28, 8.60)	0.76	−0.81 (−7.15, 5.53)	0.80
**Interest in the experiment** (ref. No)										
Yes	−0.47 (−1.09, 0.14)	0.13	0.26 (0.02, 0.49)	0.03	0.89 (−8.29, 10.06)	0.85	1.53 (−7.40, 10.46)	0.74	−2.00 (−9.62, 5.62)	0.61
**Bad news received** (ref. No)										
Yes	0.55 (0.15, 0.96)	0.01*	0.25 (0.10, 0.41)	< 0.01*	0.78 (−5.23, 6.79)	0.80	0.68 (−5.17, 6.54)	0.82	1.63 (−3.37, 6.62)	0.52
Category 6: Perceived classroom conditions during the test										
**Noise in classroom** (ref. No)										
Yes	−0.01 (−0.05, 0.03)	0.58	0.11 (−0.03, 0.25)	0.12	−3.32 (−8.82, 2.19)	0.24	0.95 (−4.41, 6.31)	0.73	−4.83 (−9.40, −0.26)	0.04*

Note: CI, confidence interval; HRT−SE, hit reaction time standard error. Model adjusted for: sex, age, country of birth, PMA−R, maternal occupational social class and poverty. Associations are estimated using conditional linear regression with school as strata. Association reported as additive change in the mean of outcome. * statistical significance at the 0.05 level.
